# Divide and Conquer—Targeted Therapy for Triple-Negative Breast Cancer

**DOI:** 10.3390/ijms26041396

**Published:** 2025-02-07

**Authors:** Milica Nedeljković, Ana Vuletić, Katarina Mirjačić Martinović

**Affiliations:** Department of Experimental Oncology, Institute for Oncology and Radiology of Serbia, 11000 Belgrade, Serbia; radovanovica@ncrc.ac.rs (A.V.); katarina.mirjacic@ncrc.ac.rs (K.M.M.)

**Keywords:** triple-negative breast cancer, molecular subtypes, androgen receptor, TGF-β pathway, notch pathway, Wnt/β-catenin pathway, JAK/STAT pathway, PI3K/AKT pathway, EGFR, VEGFR, immunotherapy, immune checkpoint inhibitors, PARP inhibitors

## Abstract

Triple-negative breast cancer (TNBC) is the most aggressive and malignant type of breast cancer with limited treatment options and poor prognosis. One of the most significant impediments in TNBC treatment is the high heterogeneity of this disease, as highlighted by the detection of several molecular subtypes of TNBC. Each subtype is driven by distinct mutations and pathway aberrations, giving rise to specific molecular characteristics closely connected to clinical behavior, outcomes, and drug sensitivity. This review summarizes the knowledge regarding TNBC molecular subtypes and how it can be harnessed to devise tailored treatment strategies instead of blindly using targeted drugs. We provide an overview of novel targeted agents and key insights about new treatment modalities with an emphasis on the androgen receptor signaling pathway, cancer stem cell-associated pathways, phosphatidylinositol 3-kinase (PI3K)/AKT pathway, growth factor signaling, and immunotherapy.

## 1. Introduction

Breast cancer is the most common malignancy and the leading cause of cancer-related mortality in women globally [1]. Triple-negative breast cancer (TNBC) is characterized by the absent expression of estrogen (ER) and progesterone (PR) receptors and lack of human epidermal growth factor receptor 2 (HER-2) overexpression/amplification. Of the newly diagnosed breast cancer cases, 10–20% will be TNBC [2]. This disease is associated with more frequent metastatic spread and poorer overall survival in comparison to non-TNBC breast tumors [3].

TNBC seems intrinsically more aggressive compared to other breast cancer subtypes. Moreover, TNBC often becomes resistant to chemotherapy and does not respond to the workhorses of breast cancer treatment—endocrine and HER2 targeted therapy—making clinical management challenging [4].

Another hurdle impeding successful treatment is TNBC’s high molecular heterogeneity. TNBC is an umbrella term that includes several entities with distinct clinical behavior and drug sensitivity. Our extensive review of TNBC chemoresistance emphasized the importance of patient stratification according to genomic biomarkers for improving treatment outcomes [4]. Personalized therapy remains an elusive ideal, but treatment modality tailored to specific TNBC subtypes is an achievable goal that could revolutionize TNBC management.

This review will delve into TNBC molecular heterogeneity and focus on treatment implications. We will concentrate on recent data and explore novel therapeutics, especially immunotherapy.

## 2. TNBC’s Molecular Heterogeneity

Determination of molecular subtypes of TNBC is a work in progress set in motion by milestone research by Lehmann et al. [5]. Based on an in-depth analysis of TNBC gene expression profiles, the authors inferred the existence of six subtypes: basal-like 1, basal-like 2, luminal androgen receptor (LAR), mesenchymal, mesenchymal stem-like (MSL), and immunomodulatory [5]. In a follow-up study, the subtypes were condensed into four: basal-like 1 (BL1), basal-like 2 (BL2), mesenchymal (MES), and LAR [6]. The authors subsequently established that the immunomodulatory gene expression profile arose from the tumor-infiltrating lymphocytes (TILs) and stromal mater instead of the tumor bulk [6]. Nevertheless, another study included immunomodulatory as one of the molecular subtypes of TNBC. Bareche et al. proposed that TNBCs be stratified into five subtypes: BL1, immunomodulatory, MES, LAR, and MSL [7]. The authors obtained copy number variation (CNV), gene expression, and somatic mutation data for 550 TNBC samples from the Cancer Genome Atlas and Molecular Taxonomy of Breast Cancer International Consortium (METABRIC). They used the TNBCtype online subtyping tool based on the Lehmann et al. study to bioinformatically analyze the data [7]. The five proposed subtypes displayed distinct clinical and pathological characteristics [7]. Burstein et al. analyzed RNA and DNA profiles of almost 200 TNBC samples and established four distinct signatures of 80 genes [8]. The identified stable subtypes were named basal-like immune activated (BLIA), basal-like immune suppressed (BLIS), LAR, and MES [8]. Prognosis differed significantly between the subtypes, and each subtype showed a specific motif of gene expression, pathway aberrations, and CNV [8]. The classification by Burstein et al. displayed a better correlation between molecular subtype and prognosis compared to the previous studies.

Jiang et al. analyzed a cohort of 465 TNBCs and, based on genomic and transcriptomic profiles, subtyped the tumors into LAR, immunomodulatory, BLIS, and MES subtypes [9]. The same authors developed an immunohistochemistry (IHC) method to stratify TNBCs [10]. Recently, a new investigation validated the use of IHC as a surrogate to subclassify TNBC [11]. Liu et al. stratified 165 TNBC tumors based on the whole-transcriptome expression analysis [12]. The authors not only analyzed mRNA expression but also the expression of long-non-coding RNAs (lncRNAs), which are now known to be crucial in many pathways and potentially may serve as biomarkers in cancer [12,13]. The four subtypes were classified as immunomodulatory, LAR, MES, and BLIS [12]. Another study that used transcriptome profiling proposed three subtypes of TNBC, C1–C3, of which C2 displayed properties similar to BLIS and C3 to BLIA [14]. The C1 subtype was composed of TNBCs featuring a molecular apocrine phenotype [14].

Alternative approaches in classifying TNBCs have been attempted in recent years, owing to the availability of new technologies and new types of data. Wang et al. identified four subtypes based on alternative polyadenylation events, a posttranscriptional modification frequently occurring during tumor development [15]. Other strategies centered on alternative splicing [16] and DNA methylation [17]. Several studies utilized metabolic pathways to stratify TNBCs into subtypes with distinct metabolic features and significant clinical differences [18,19]. The tumor microenvironment (TME) was utilized to subtype TNBC tumors based on their immunogenomic profile [20]. Another study used the presence of TILs and metabolic markers to subdivide TNBCs [21]. Moreover, it was demonstrated that molecular subtypes have specific TME profiles [22].

TNBC incidence shows variation according to race and ethnicity. In a large population study of breast cancer patients, TNBC was twice as prevalent among African American women, while Hispanic women exhibited a 30% increased likelihood of diagnosis in comparison to their non-Hispanic Caucasian counterparts [23]. Therefore, it was investigated whether molecular subtypes also demonstrate disparities among different ethnicities. Yuan Chun Ding et al. conducted one of the first studies that analyzed TNBC subtypes in relation to ethnicities: non-Hispanic Caucasian, Hispanic, African American and Asian [24]. In comparison to the average proportions across all groups, the BLIS subtype was significantly associated with Hispanic ethnicity, whereas Asians had a lesser incidence of BLIS but a significantly higher frequency of the LAR subtype [24]. Another study examined TNBC molecular subtypes in African Americans as well as patients from various regions of Africa and detected marked variations in subtype distribution [25]. BL1 tumors were more predominant in Ghanaian and Ethiopian patients, and the IM subtype was more frequent in African American patients [25]. In accordance with these results, a recent study also observed immune pathway enrichment in African American TNBC patients [26]. Determining the distributions of TNBC subtypes in relation to race and ethnicity may aid in developing more effective treatment modalities and better predictors of prognosis, especially for women of African ancestry that are more likely to be diagnosed younger and have an advanced disease. The results of these studies stress the relevance of considering genetic ancestry in the context of prognosis and treatment planning for TNBC.

**Figure 1:**
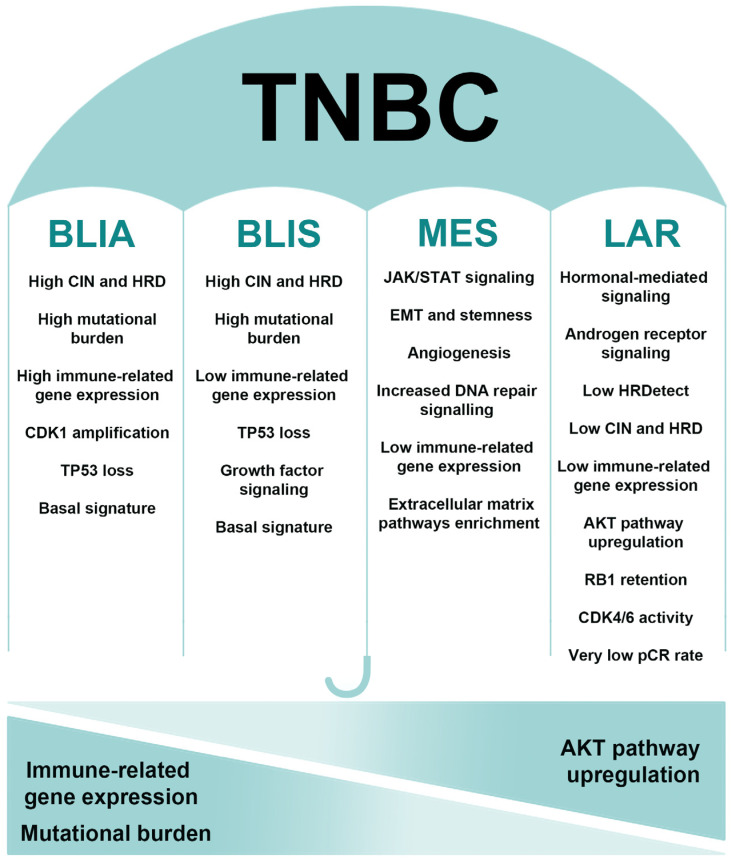
Overview of TNBC molecular subtypes and their key characteristics. Aggregated information from Lehmann et al. [6], Burstein et al. [8], Jiang et al. [9], Bareche et al. [7], Lehmann et al. [30], Asleh et al. [31], Zhao, S. et al. [10], Abbreviations: triple-negative breast cancer (TNBC); luminal androgen receptor (LAR), basal-like immune suppressed (BLIS), basal-like immune activated (BLIA), mesenchymal (MES), epithelial–mesenchymal transition (EMT), chromosomal instability (CIN), homologous recombination deficiency (HRD), pathologic complete response (pCR), cyclin-dependent kinase (CDK), Janus kinase/signal transduction and transcription activation (JAK/STAT), retinoblastoma susceptibility gene (RB1).

TNBC classification is complex and multifaceted, with no true consensus in the literature. Nevertheless, the four subtypes identified by gene expression profiles (BLIA/immunomodulatory, BLIS, LAR, and MES) are the most thoroughly validated. Illustrated in Figure 1 are the key characteristics of these four subtypes of TNBC. The efficacy of this subtyping was recently confirmed by a FUTURE-SUPER phase II clinical trial (NCT04395989), which reported encouraging results [27]. Patients who received targeted therapy according to the aforementioned TNBC subtypes had significantly longer progression-free survival compared to the control group [27]. The FUTURE-SUPER study is one of the first steps in what we think is the future direction of TNBC treatment [27]. Currently, numerous agents targeting various pathways in TNBC are being investigated in clinical trials. However, the selection of patients who can derive the most benefit from a given drug is still lacking. In the great majority of studies, patient selection is not informed by TNBC molecular subtypes. Even when patients are preselected for pathway aberrations, it can still be insufficient. For example, the CAPItello-290 phase III trial examining an inhibitor of all three AKT isoforms plus chemotherapy in advanced TNBC failed to show improved overall survival in comparison to chemotherapy plus placebo, even in the cohort with phosphatidylinositol 3-kinase (PIK3CA)/AKT1/phosphatase and tensin homolog deleted on chromosome ten (PTEN) alterations [28]. While alterations of a signaling pathway can be present in multiple TNBC subtypes, their frequency and significance for tumor survival will vary according to molecular subtype. This is why we believe stratifying patients based on the status of a single pathway is not enough, as evidenced by the mixed results of clinical trials, and why stratification should be approached in the context of molecular subtypes. The TNBC subtypes offer a greater insight into the molecular foundation of tumors. The two recent studies, FUTURE phase Ib/II (NCT03805399) and FUTURE-SUPER phase II (NCT04395989), which employed this approach, showed encouraging results, emphasizing the importance of taking into account the interdependency and interplay of molecular subtype and biomarkers [27,29].

In this review, we will focus on the BLIA, BLIS, LAR, and MES/MSL TNBC subtypes, give a brief overview of characteristics and key signaling pathways of each subtype, and attempt to connect the existing clinical trials of targeted drugs with the subtype they would be most effective at treating.

## 3. Luminal Androgen Receptor—LAR

The LAR subtype is primarily characterized by the expression of the androgen receptor and luminal expression profile [5,9]. In accordance with luminal properties, the AKT pathway is frequently upregulated in the LAR subtype. Phosphatidylinositol 3-kinase (PI3KCA) mutations are significantly more common in LAR than in other TNBC tumors [9]. Like in all TNBCs, ER protein expression is absent in LAR, and HER2 is not overexpressed. However, LAR tumors are noted for elevated expression of various genes connected with estrogen and for carrying HER2 activating mutations, which corresponds to the luminal pattern of gene expression [8,9]. LAR cells seem to be dependent on hormonal-mediated signaling [30]. LAR tumors display very limited immune-related gene expression as well as low levels of homologous recombination deficiency (HDR) and chromosomal instability (CIN) [9]. These tumors show high phosphorylation of retinoblastoma protein (RB), indicating a functional G1S checkpoint and sensitivity to cyclin-dependent kinase 4/6 (CDK4/6) inhibition [30]. In addition, cyclin-dependent kinase inhibitor 2A (*CDKN2A)* loss was more prevalent in LAR compared to other subtypes, while the reverse was true for retinoblastoma susceptibility gene (*RB1*) [9]. LAR tumors are highly chemo-resistant and, subsequently, have a very low pathologic complete response (pCR) rate [32]. Due to their high chemoresistance, LAR tumors are in urgent need of effective targeted therapy.

### 3.1. Androgen Receptor as a Treatment Target in LAR

**Figure 2:**
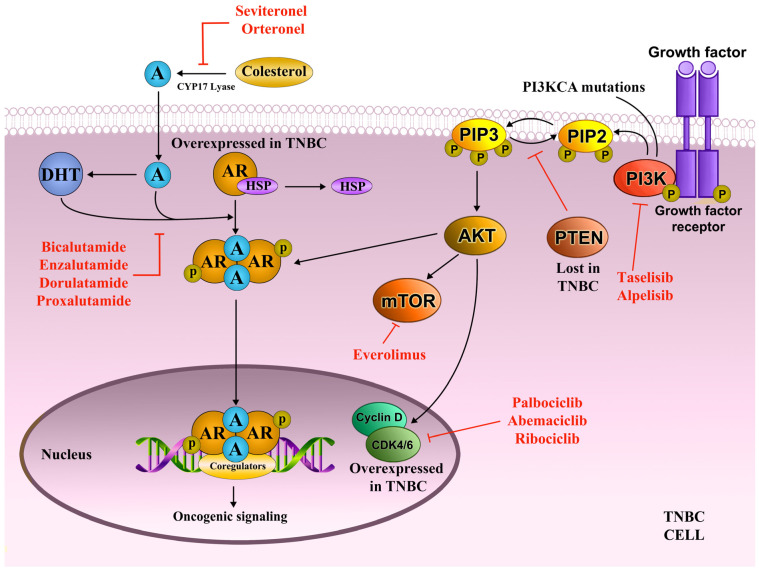
Overview of AR and associated pathways and their potential inhibitors in triple-negative breast cancer (TNBC). Inhibitors are shown in red. Androgens are synthesized from cholesterol by CYP17 lyase. CYP17-lyase inhibitors seviteronel and orteronel block the production of androgens from cholesterol. Upon entering the cell, androgens may be converted to DHT, a stronger AR agonist. Androgens activate AR, causing its release from HSP complex. AR subsequently dimerizes and translocates into the nucleus, where it modulates the transcription of target genes. Antiandrogens including bicalutamide, enzalutamide, darolutamide and proxalutamide, inhibit androgen binding to AR. The PI3K/AKT/mTOR and AR signaling pathways have significant crosstalk. Activation of AKT induces upregulation of AR through several mechanisms. Taselisib and alpelisib inhibit PI3K, therefore inhibiting AKT activation. AR also regulates the cell cycle. After the binding of a ligand, AR induces the translation and accumulation of D-type cyclins and their associated CDKs. Palbociclib, abemaciclib and ribociclib inhibit the CDK4/6. Abbreviations: triple-negative breast cancer (TNBC); androgen receptor (AR); androgen (A); dihydrotestosterone (DHT); heat shock protein (HSP); phosphoinositide 3-kinase (PI3K); mammalian target of rapamycin (mTOR); phosphatidylinositol-3,4,5-trisphosphate (PIP3); phosphatase and tensin homolog deleted on chromosome ten (PTEN).

Inactive androgen receptor (AR) is found in the cytoplasm in a complex with heat shock and other chaperone proteins (HSP-70, HSP-90) [33]. The binding of androgen causes a conformational change leading to AR activation and the translocation of the newly formed AR homodimer into the nucleus, where it recruits coregulators and induces transcription of target genes (Figure 2) [33]. Another, more rapid method of AR activation relies on AR interaction with mitogen-activated protein kinase (MAPK)/extracellular signal-regulated kinase (ERK) signaling cascade, resulting in cell proliferation [33].

Preclinical studies demonstrated that LAR cells are dependent on androgen signaling and more sensitive to anti-androgen therapy [30,34]. Collectively, these findings provided a rationale for targeting the AR pathway.

Gucalp et al. conducted the first clinical trial (NCT00468715) for the treatment of metastatic breast cancer utilizing anti-androgen therapy (Table 1) [35]. The study included twenty-six patients who received AR antagonist bicalutamide. The primary endpoint was clinical benefit rate (CBR), which reached 19% at 6 months. The intervention was well tolerated by the participants [35]. Following these encouraging results, a number of phase II and III clinical trials investigated the effects of bicalutamide, alone or in conjunction with chemotherapy in metastatic TNBC (NCT02348281, NCT02353988, NCT03055312). However, none of the trials reported results and all were prematurely terminated.

When second-generation AR inhibitors (enzalutamide, dorulatamide) were developed for use in metastatic castrate-resistant prostate cancer, it was speculated that they may prove beneficial in TNBCs expressing AR. A phase II clinical study (NCT01889238) investigated enzalutamide in patients with locally advanced or metastatic AR-dependent TNBC and showed clinical activity of the drug [36]. Enzalutamide was given as monotherapy. The primary endpoint of the trial, CBR at 16 weeks, was 25% in the intent-to-treat population and 33% in the evaluable subgroup (patients that had at least one post-baseline assessment available). The treatment was well tolerated [36]. Based on the satisfactory performance of enzalutamide in advanced tumors, the feasibility of treatment for early-stage AR-positive TNBC was also evaluated (NCT02750358). The primary endpoint of the study was treatment enrollment at the one-year mark, defined as the rate of discontinuation of participation due to tolerability-related events. Of the 50 patients who entered the trial, 35 completed one year of treatment and 32 chose to continue the treatment for a subsequent year [37]. The study demonstrated that enzalutamide is a sound and well-tolerated treatment approach in low-stage AR+ TNBC. However, no data on the effect of this treatment regimen on the prognosis were posted [37]. Still, these results set the stage for researchers to assess the utility of targeting the AR pathway in the treatment of non-metastatic TNBC. A phase II study (NCT02689427) demonstrated the efficacy of neoadjuvant enzalutamide together with paclitaxel for increasing the customarily low pCR rate of LAR-enriched TNBC. Of the twenty-four enrolled patients, 42% achieved pCR with a satisfactory safety profile of the utilized drug regimen. The results demonstrate good clinical activity of enzalutamide in a neoadjuvant setting [38]. A phase II trial evaluating enzalutamide as monotherapy and enzalutamide in combination with mifepristone or chemotherapy in patients with advanced TNBC is in progress, currently recruiting participants (NCT06099769). Anti-androgen proxalutamide was investigated in a phase I trial (NCT04103853) on metastatic breast cancer patients including 14 AR+ TNBC which had 38.5% CBR at 8 weeks [39]. A randomized phase II study investigated dorulatamide in comparison to capecitabine in a cohort of advanced AR+ TNBC (NCT03383679). Dorulatamide performed worse than the control with a CBR of 16 weeks of 24.5% compared to capecitabine’s 47.8% [40].

Another approach is to use androgen biosynthesis inhibitors, agents such as seviteronel and orteronel. Both sviteronel and orteronel inhibit cytochrome P450 17α-hydroxylase/17,20-lyase (CYP17 lyase), an enzyme essential for androgen synthesis and therefore lower circulating androgens. Seviteronel exhibited 33% CBR at 16 weeks in patients with advanced AR+ TNBC in a phase I study (NCT02580448) [41]. Currently, a phase II study is recruiting participants in order to test seviteronel in combination with docetaxel in AR + metastatic TNBC (NCT04947189). On the other hand, orteronel showed poor clinical activity as monotherapy in a phase II study (NCT01990209) in AR expressing advanced breast cancer including a TNBC cohort. Further development was discontinued [42]. An overview of the AR pathway and representative targeted agents are shown in Figure 2.

### 3.2. AKT Pathway and Cell Cycle as Treatment Targets in LAR

Anti-androgens as monotherapy or in conjunction with chemotherapy showed mixed results. Targeting other cancer driver pathways in combination with anti-androgens could be a useful strategy in LAR TNBCs.

The AKT pathway is one of the crucial mechanisms that control cell proliferation, growth, survival, and motility [43]. PI3K transduces growth factor signaling and activates the AKT kinase, which results in the phosphorylation of the mammalian target of rapamycin (mTOR), leading to increased protein synthesis and cell growth [43]. AKT activity is downregulated by the tumor suppressor PTEN. Activated AR can interact with the regulatory subunit of PI3K, leading to AKT pathway upregulation. LAR tumors commonly carry PI3KCA mutations, indicating the significance of the crosstalk between AR and AKT pathways (Figure 2) [44].

Concurrent targeting of AR and PI3K was investigated in a phase I/II trial testing enzalutamide and taselisib, a selective class I PI3K inhibitor (NCT02457910). Even with early termination, the regimen had increased CBR in AR+ TNBCs compared to enzalutamide as monotherapy, especially in participants with LAR tumors, as confirmed by RNA expression. In general, TNBC patients achieved a 16-week CBR of 35.7% and median progression-free survival (PFS) of 3.4 months. CBR rate was highest in LAR tumors compared to non-LAR (75.0% vs. 12.5%) [45]. A phase I trial (NCT03207529) investigated enzalutamide in combination with the alfa-subunit selective PI3K inhibitor alpelisib in patients with AR- and PTEN-positive advanced breast cancer, including TNBC. The study concluded on 31 December 2023; however, no results have been posted as of yet. A phase III trial is currently recruiting participants in order to study everolimus, a protein kinase inhibitor of the mTOR, in LAR TNBCs (NCT05954442).

The AR pathway has a key role in regulating cell cycle progression in TNBC [46]. AR activation has been shown to increase levels of cyclin D1, while AR expression correlated with RB expression [47,48]. LAR tumors were demonstrated to have highly phosphorylated RB. [30] Moreover, substantial crosstalk exists between the AKT and CDK4/6 pathways in breast cancer, with PI3K activity shown to increase cyclin D1 levels [49].

In the canonical model of cell cycle entry, cyclin D1 binds to and activates CDK4/6, which then proceeds to phosphorylate RB, resulting in the dissolution of the RB/E2F complex. The freed transcription factor E2F in turn induces the transcription of genes that drive cell cycle progression [50].

In pre-clinical research, LAR cell lines displayed high sensitivity to CDK4/6 inhibitors both in vitro and in vivo [51]. In addition, palbociclib, a selective inhibitor of CDK4/6, already demonstrated good efficacy in two large phase III trials on advanced ER+ breast cancer (NCT01740427 and NCT01942135). Therefore, it was hypothesized that combined targeting of CDK4/6 and AR pathways can maximize the inhibition of RB phosphorylation and provide the means to effectively counter LAR tumors. This premise is being investigated in several ongoing clinical trials. A combination of bicalutamide with palbociclib showed preliminary activity in metastatic AR+ TNBC with a 33% CBR at 6 months (NCT02605486) [52]. The PAveMenT clinical trial is currently recruiting participants in order to investigate palbociclib and avelumab (PD-L1 blocking human IgG1 lambda monoclonal antibody) in metastatic AR+ TNBC (NCT04360941) [53]. Abemaciclib and ribociclib, also potent CDK4/6 inhibitors, are currently being tested in early-phase clinical trials in advanced AR+ TNBC patients (NCT06365788, NCT05095207, NCT03090165).

Although antiandrogens are, as of yet, not approved for use in clinical practice for the treatment of TNBC, in some instances, they were prescribed outside of the clinical trial setting. A retrospective analysis of the antiandrogens’ efficacy in locally advanced or metastatic AR+ TNBC, the “real-life data”, as termed by the authors, showed findings corresponding to the results of clinical trials [54].

It is clear that AR targeting holds much potential for LAR treatment, but crucial issues will have to be addressed before it can be fully realized. One of the underpinning causes behind mixed results of clinical trials could be the lack of consensus on how AR positivity is defined. IHC is considered a gold standard for determining AR expression. However, different studies use varying cut-off points—from 1% to 30%, with most using 10% as a measure of AR expression (Table 1). This makes comparing results difficult. A more significant issue is that AR expression is often used as a surrogate to define the LAR subtype. In numerous studies, the terms AR-positive TNBC and LAR are used interchangeably; however, these phrases are not synonyms. Lim, B. et al. have demonstrated that only 70% AR positivity, as defined by IHC, corresponds to the LAR subtype [38]. In another study, LAR tumors, as defined by gene expression profiles, showed a much more favorable response to anti-AR therapy [45]. It is clear that the stratification of patients needs to be improved in future studies, as it remains key for maximizing the efficacy and utility of novel targeted therapies.

## 4. Mesenchymal and Mesenchymal Stem Cell-Like—MES/MSL

The MES/MSL subtype displays the characteristics of epithelial–mesenchymal transition (EMT) and breast cancer stem cells (CSCs) and is therefore enriched in pathways crucial for the maintenance of these phenotypes: transforming growth factor beta (TGF-β), Notch, Wnt/β-Catenin and Janus kinase/signal transduction and transcription activation (JAK/STAT) pathways, showing the possibility of utilizing this axis for targeted therapy [5,7,9]. We have previously thoroughly reviewed the key role of CSCs and the associated pathways in TNBC development, especially chemoresistance [4]. Strategies targeting developmental pathways and CSCs may be the most worthwhile approach to treating MES.

**Figure 3:**
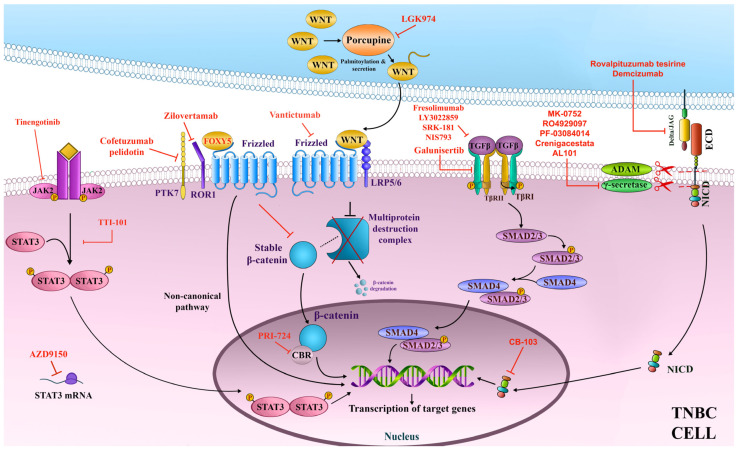
Overview of cancer stem cell-associated pathways and their potential inhibitors in triple-negative breast cancer (TNBC). Inhibitors are shown in red. From left to right: The mammalian JAK/STAT signaling pathway is made up of four proteins featuring the Janus kinase domain: JAK1-3 and TYK2, along with seven proteins belonging to the STAT family. JAKs are cytoplasmic proteins and are linked to transmembrane receptors. Binding of an extracellular ligand initiates the trans-phosphorylation of JAKs, which subsequently phosphorylate STAT monomers, which then migrate to the nucleus and regulate the transcription for target genes. Tinengotinib is a selective small-molecule kinase inhibitor, which inhibits JAK 1/2 and Aurora/STAT3 pathways. TTI-101 is small-molecule kinase inhibitor targeting STAT3. AZD9150 is a novel antisense nucleotide inhibitor of STAT3. In the Wnt canonical pathway, the degradation complex rapidly eliminates β-catenin in the absence of Wnt. When Wnt binds to its receptor Frizzled and the co-receptors LRP5/6, the destruction complex is disrupted, leading to the stabilization of β-catenin, which then translocates to the nucleus and activates the transcription of target genes. In order for Wnt to bind Frizzled, palmitoleoylation on a hairpin 2 motif by the O-acyltransferase Porcupine is necessary. LGK974 is a specific small-molecule inhibitor of Porcupine. PRI-724 is a novel modulator of Wnt signaling that prevents the interaction of CREB binding protein with β-catenin. Vantictumab is a mAb that binds to frizzled receptors and neutralizes canonical WNT signaling. Zilovertamab is a mAb against ROR1, a protein that stimulates the activity of the non-canonical Wnt signaling. Protein tyrosine kinase 7 is a Wnt pathway co-receptor and is a target of the antibody–drug conjugate cofetuzumab pelidotin. Foxy-5 is a peptide mimic of the WNT5A, designed to reconstitute lost WNT5A signaling, since WNT5A seems to reduce migration and invasion of cancer cells. Activation of the TGF-β pathway occurs when TGF-β interacts with its type II receptor, leading to the formation of a complex with the type I receptor. This complex subsequently recruits SMAD2 and SMAD3, which are phosphorylated and form a heteromeric complex with SMAD4. The resulting complex translocates into the nucleus of a tumor cell, where it activates the transcription of target genes. Fresolimumab, a human mAb that neutralizes all three isoforms of TGF-β. SRK-181 is a TGF-β1 mAb. NIS793 is a human anti-TGF-β1 IgG2 mAb. LY3022859 is an anti-TGFβRII IgG mAb. Galunisertib is a small molecule inhibitor of the TGF-β receptor I. Comprising four cell surface receptors and five transmembrane ligands, the Notch signaling pathway necessitates direct cellular contact for its activation. When a Notch ligand attaches to a receptor on a neighboring cell, it initiates the release of the receptor’s intracellular domain—NICD. This release is accomplished through a series of cleavages carried out by ADAM proteases and γ-secretase. Once released, NICD transmits into the nucleus, where it regulates transcription of target gene. MK-0752, RO4929097, PF-03084014, crenigacestata and AL101 are γ-secretase inhibitors. Rovalpituzumab tesirine is an antibody–drug conjugate targeting one of the transmembrane ligands DLL3. Demcizumab is an anti-DLL4 mAb. CB-103 is an agent that prevents the released Notch receptor NICDs from activating transcription of target genes. Abbreviations: Janus kinase/signal transducer and activator of transcription (JAK/STAT); low-density lipoprotein receptor-related proteins (LRP5/6); protein tyrosine kinase 7 (PTK7); transforming growth factor beta (TGF-β); suppressor of mother against decapentaplegic (SMAD); Notch receptor intracellular domain (NICD); Notch receptor extracellular domain (ECD); monoclonal antibodies (mAbs).

In addition, MES tumors are characterized by upregulation of growth factor signaling (EGFR, PDGF), high phosphorylation of ATR and ATM, and enhanced DNA repair signaling [5,30]. In regards to the mutational burden, MES tumors are situated in between LAR and BLIS/BlLIA subtypes [9]. However, the MES mutational profile has proven challenging to define, containing a mix of signatures. MES tumors demonstrated global DNA hypomethylation, excluding regions containing immune-related genes that are hypermethylated [30]. Consequently, MES tumors have low expression of interferon-gamma (IFN-γ), immune checkpoint, and antigen presentation genes [30]. It seems MES tumors possess immune-evasive mechanisms, making them unlikely to respond to immunotherapy. These tumors frequently carry aberrations of the PI3K/AKT/mTOR pathway [9]. MES and MSL subtypes are also enriched for genes involved in angiogenesis [5,7]. In the following sections, we will discuss agents directed against signaling pathways crucial for the maintenance of MES/MSL tumors. Figure 3 showcases crucial targets and the corresponding agents in the context of developmental pathways.

### 4.1. TGF-β Pathway

TGF-β has three isoforms and belongs to a large cytokine superfamily that includes numerous growth factors [55]. The pathway is activated when TGF-β binds to its corresponding type II receptor, causing it to form a complex with the type I receptor. Suppressor of mother against decapentaplegic (SMAD) SMAD2 and SMAD3 are then recruited by the TGF-β receptor complex, phosphorylated, and subsequently form a heteromeric complex with SMAD4 [55]. The heteromeric complex translocates into the nucleus of a tumor cell and activates the transcription of target genes, which promote EMT, growth, proliferation, and metastatic spread (Figure 3) [56]. In addition, TGFβ is capable of modulating TME to enhance angiogenesis and suppress the anti-tumor activity of the immune system [57]. TGFβ is able to activate several noncanonical pathways, such as NF-κB, AKT, and ERK, among others [57].

Targeting the TGF-β pathway utilizes anti-TGF-β monoclonal antibodies (mAbs), small-molecule inhibitors aimed at the TGF-β receptor, and ligand traps that prevent the TGFβ–receptor interaction (Table 2).

Fresolimumab, a human mAb that neutralizes all three isoforms of TGF-β, was investigated in a clinical trial in metastatic breast cancer but displayed subpar results (NCT01401062). SAR439459 is another human mAb that neutralizes TGF-β1,2,3. This mAb was evaluated in two trials (NCT03192345, NCT04729725) in advanced solid tumors; however, both were terminated due to unclear efficacy and unacceptable toxicity profile [58]. A trial investigating SRK-181, a TGF-β1 mAb in solid tumors, is currently recruiting participants (NCT04291079). A phase Ib trial of NIS793 (a human anti-TGF-β1 IgG2 mAb) in combination with a checkpoint inhibitor spartalizumab, showed preliminary activity, and the regimen was well tolerated in patients with advanced solid tumors, including breast cancer (NCT02947165) [59]. A phase I clinical trial of LY3022859, an anti-TGFβRII IgG mAb, was conducted for patients with advanced solid tumors but the treatment was deemed unsafe due to uncontrolled cytokine release, and further research was discontinued (NCT01646203) [60]. Vigil (gemogenovatucel-T) is an advanced vaccine created using autologous tumor tissue with threefold action. Vigil is a bifunctional short-hairpin RNA that reduces the expression of TGF-β1/β2 and furin. It is transfected into autologous tumor cells using a plasmid encoding the human granulocyte macrophage colony stimulating factor (*GMCSF*) gene. As a result, the tumor cells are modified to reduce tolerance escape, enhance tumor-specific antigen presentation, and increase dendritic cell activation. Vigil in combination with PD-L1 inhibitor durvalumab was tested in a pilot study (NCT02725489) in advanced TNBC, displaying a good safety profile and encouraging preliminary efficacy [61].

A phase I study (NCT02672475) assessing the safety and optimal dosage of galunisertib (a small-molecule inhibitor of the TGF-β receptor I) in combination with chemotherapy in metastatic AR-negative TNBC was recently completed; however, no results were posted.

TGFβ ligand traps are fusion proteins rationally designed to neutralize TGFβs. Bintrafusp alfa trap is a bifunctional fusion protein composed of the ectodomain of the human TGF-β receptor II connected with a heavy chain of a human IgG1 mAb blocking PD-L1. Several trials were conducted evaluating bintrafusp alfa in TNBC. A phase II clinical trial (NCT04489940) investigated the effectiveness of bintrafusp alfa as a single agent in TNBC treatment, while a phase Ib trial (NCT04296942) tested bintrafusp alfa in combination with BN-brachyury recombinant vector-based cancer vaccine in order to determine the overall response rate and safety of the intervention. Both trials were terminated, the former because the probability of success was too low for the current competitive environment, the latter due to slow accrual. Another phase Ib trial (NCT03579472) determining recommended dosage and asserting the safety and tolerability of bintrafusp alfa together with eribulin mesylate in the treatment of metastatic TNBC was also terminated and further development of the agent discontinued. A phase I/II study (NCT04789668) assessed the side effects and recommended dose of bintrafusp alfa together with pimasertib (a selective MEK1/2 inhibitor), as well as overall survival of patients with metastatic TNBC; however, no results have been posted as of yet.

Significant hurdles stand in the way of TGF-β-based targeted therapy for MES/MSL TNBC. There are concerns with inadequate selectivity and/or specificity of inhibitors leading to toxicity, since TGF-β is crucial for maintaining tissue homeostasis. The cost of mAb production is high, mAb dissemination within tumor bulk may be insufficient and the availability of the TGF-β to mAb can be low [62]. Upregulated TGFβ pathway activity was connected with the failure of immune checkpoint inhibitors, providing a rationale for combining TGFβ neutralization with immune checkpoint inhibition in cancer treatment. The design of bintrafusp alfa, while efficient, also restricts TGFβ inhibition to the locations of PD-L1 within tumor tissue and may potentially weaken the neutralization of active TGFβ. Using independent agents may be preferable to bintrafusp alfa. Moreover, there are indications that the TGF-β pathway serves as a tumor suppressor in early-stage breast cancer and care must be taken when targeting TGF-β not to inhibit the tumor-suppressive role [55]. Figure 3 shows the TGF-β pathway with crucial targets and the corresponding inhibitors.

### 4.2. Notch Pathway

The Notch signaling pathway encompasses four cell surface receptors and five transmembrane ligands. The activation of Notch is contingent upon direct contact between cells. When a Notch ligand binds to a receptor on a neighboring cell, it triggers the release of the receptor’s intracellular domain (NICD). This mechanism is enabled by a series of cleavages performed by ADAM proteases and γ-secretase. Subsequently, NICD moves into the nucleus, where it plays a crucial role in regulating the transcription of target genes (Figure 3) [63].

The altered Notch signaling pathway has a wide array of effects in human tumors and is implicated in all phases of tumor development, from tumorigenesis to progression and metastasis. Aberrations of the Notch pathway are connected to all major characteristics of cancer, including evasion of the immune response, modulation of the immune microenvironment, EMT, maintenance of CSCs, angiogenesis, energy metabolism, growth, metastasis, and, especially relevant for TNBC, chemotherapy resistance [63,64]. Notch receptors 1 to 4 are closely associated with resistance to chemotherapy [65,66]. Notch activation may lead to poor prognosis and relapse in breast cancer [67]. *Notch* mutations are abundant in TNBC and are a potential oncogenic driver [68]. Furthermore, continuous Notch-3 signaling may drive oncogenic activities within the basal subtype of TNBC [69].

The fundamental role of Notch signaling in tumor development has spurred the exploration of the Notch pathway as a target of novel therapeutics. Neutralizing γ-secretase activity with inhibitors (GSIs) is the main strategy. Evidence from preclinical studies indicates that these inhibitors effectively increase the susceptibility of TNBC cell lines to chemotherapy, prompting clinical trials to evaluate GSIs as monotherapy and in combination with chemotherapeutic and other targeted agents for TNBC treatment (Table 3).

MK-0752, a GSI, was investigated in two phase I trials in advanced breast cancer as a single treatment (NCT00106145) and in combination with docetaxel (NCT00645333), displaying acceptable tolerability and preliminary efficacy [70,71]. Of note, after undergoing several treatment cycles with MK-0752, patients exhibited a diminished burden of CSC [70]. RO4929097 GSI was evaluated in three phase I/II trials on advanced TNBC as monotherapy (NCT01151449), in conjunction with chemotherapy (NCT01238133), and in combination with vismodegib (NCT01071564), an agent targeting the Hedgehog pathway. All three trials were terminated, but RO4929097 with chemotherapy showed preliminary activity in TNBC [72]. The therapeutic regimen involving PF-03084014 GSI and docetaxel was well tolerated and demonstrated clinical efficacy in patients with advanced TNBC (NCT01876251) [73]. The side effects and efficacy of the same GSI as monotherapy for advanced TNBC carrying genomic aberrations of *NOTCH* receptors were assessed in the phase II study (NCT02299635). A small cohort of patients without genomic aberrations of NOTCH was also included in the trial. However, the study was prematurely terminated by the sponsor due to changes in the strategy of PF-03084014 development. There were no safety concerns. PF-03084014 was also used in a biomarker study among TNBC patients with residual disease following anthracycline- and taxane-based chemotherapy treatment (NCT02338531). The study aimed to evaluate the capacity of PF-03084014 to alter the Notch pathway by downregulating the expression of *HES4*. Again, the trial was withdrawn due to the discontinuation of the development of PF-03084014. A phase I clinical trial of crenigacestata, a GSI, in conjunction with several other anticancer agents, was conducted for patients with advanced solid tumors, including breast cancer, but the treatment had low efficacy and was deemed unsafe (NCT02784795) [74]. Another GSI, AL101, was evaluated in a phase II clinical trial as a single treatment in recurrent or advanced TNBC with activated Notch but it was terminated by the sponsor’s decision (TENACITY, NCT04461600).

Novel treatment strategies are considering other components of the Notch pathway in addition to γ-secretase. Notch ligands (Delta-like 1,3,4 and JAGGED-1,2) and receptors (NOTCH 1–4) can be targeted by mAbs, ensuring greater specificity and lower toxicity compared to GSIs. Rovalpituzumab tesirine is an antibody–drug conjugate targeting DLL3, one of the transmembrane ligands of the Notch pathway. A phase I clinical trial investigating rovalpituzumab tesirine combined with other antitumor agents in advanced solid tumors, including breast cancer, was recently completed, with acceptable levels of toxicity and promising efficacy (NCT03000257). Demcizumab, an anti-DLL4 mAb, was assessed in a number of studies in solid tumors, both as monotherapy and in conjunction with other agents, showing mixed results (NCT01189968, NCT02722954, NCT01952249, NCT02259582, NCT01189929).

Another approach seeks to prevent released Notch receptor NICDs from activating transcription of target genes through the disruption of their interaction with co-activators and transcriptional factors, thereby preventing the formation of the Notch transcription complex. CB-103 is an agent with such a mechanism of action. It has been evaluated in several recent clinical trials in advanced solid tumors, including breast cancer and hematological malignancies (NCT04714619, NCT03422679, NCT05774899, NCT05464836) with mixed results. CB-103 was generally well tolerated but had limited clinical utility as monotherapy [75,76]. Figure 3 shows the Notch pathway with crucial targets and the corresponding inhibitors.

### 4.3. Wnt/β-Catenin Pathway

Wnt signaling is dysregulated in many cancers and plays a critical role in the processes of tumor initiation, preservation of stem cell characteristics, and the dissemination of cancer cells. Presently, two canonical and two β-catenin-independent models are recognized in the literature. In the main canonical model, the multi-protein destruction complex swiftly degrades β-Catenin if Wnt is not present. The engagement of Wnt with its receptor Frizzled and co-receptors low-density lipoprotein receptor-related proteins (LRP5/6) results in the disruption of the destruction complex, thereby stabilizing β-catenin. The stabilized β-catenin is then able to move into the nucleus, where it initiates the transcription of genes responsive to Wnt signaling (Figure 3).

Aberrant Wnt/β-catenin signaling has a crucial role in TNBC and is a driver of tumorigenesis. In murine models, TNBC cells in which β-catenin was knocked down showed significantly diminished migration, weaker growth, and produced markedly smaller tumors [77]. TNBCs with deregulated Wnt/β-catenin signaling had a higher propensity for the development of distant metastases [78]. Wnt/β-catenin pathway over activation was linked with poor outcomes in TNBC patients [79]. MES/MSL tumors are enriched in a number of EMT-connected genes, which are modulated by Wnt/β-Catenin pathway activity [5]. The pivotal involvement of Wnt/β-catenin signaling in the tumorigenesis of TNBC is well-established, which makes it a compelling candidate for targeted therapy. A number of Wnt/β-catenin inhibitors and modulators showed excellent activity in TNBC cell lines and xenograft models, paving the way for clinical investigations [80,81,82].

A phase I trial of LGK974 (a specific and highly effective small-molecule inhibitor of Porcupine, which is necessary for the processing of Wnt ligand secretion) as monotherapy and in combination with a checkpoint inhibitor spartalizumab displayed manageable toxicity and showed limited preliminary activity in patients with advanced solid tumors, including breast cancer (NCT01351103) [83] (Table 4).

A phase I clinical study of PRI-724 (a novel modulator of Wnt signaling that prevents the interaction of CREB binding protein with β-catenin) as a single agent was conducted for patients with advanced solid tumors, including breast cancer, and demonstrated an acceptable safety profile (NCT01302405) [84]. However, the trial was terminated due to the slow accrual of patients.

WNT5A is a β-catenin-independent ligand with a dual role in cancer, both oncogenic and tumor-suppressive [85]. In breast cancer, WNT5A seems to reduce migration and invasion of cancer cells and its loss of expression has been associated with a worse clinical course [85]. Therefore, restoring WNT5A signaling might be beneficial. Foxy-5 is a peptide mimic of WNT5A, designed to reconstitute lost WNT5A signaling. Foxy-5 was evaluated in two phase I studies as monotherapy in metastatic breast cancer, which served to determine the proper dosage and showed that the agent has low toxicity (NCT02020291, NCT02655952). Building on these results, an optimized phase II study has recently been launched to investigate the efficacy of Foxy-5 and showcase a reduced likelihood of metastasis in colon cancer patients (NeoFox, NCT03883802).

A phase I study was conducted to test the toxicity and preliminary efficacy of vantictumab, a mAb that binds to frizzled receptors and neutralizes canonical WNT signaling, in locally advanced or metastatic HER2-negative breast cancer, including a TNBC cohort (NCT01973309). Vantictumab was administered in combination with paclitaxel and had an acceptable safety profile and encouraging preliminary activity [86]. However, of concern was the incidence of bone fractures, which may prove detrimental to further clinical development of vantictumab [86]. In a similar vein, zilovertamab was investigated in a phase I trial in conjunction with paclitaxel in heavily pre-treated advanced HER2-negative breast cancer, including a TNBC group (NCT02776917). Zilovertamab is a mAb against ROR1 which is expressed by cells of numerous solid tumors, including breast cancer, but is absent from most normal postnatal tissues, making ROR1 a good target for treatment. ROR1 stimulates the activity of a variety of pathways, including the non-canonical Wnt signaling. The combined regimen of zilovertamab and paclitaxel was generally well tolerated, while efficacy evaluation results, with partial response in 6/16 (38%) or stable disease in 6/16 (38%) patients, support further development [87].

Protein tyrosine kinase 7 (PTK7) is a Wnt pathway co-receptor present on the cell surface of numerous tumors. The PTK7 protein is a target of the antibody–drug conjugate cofetuzumab pelidotin, enabling it to deliver a payload of auristatin microtubule inhibitor to tumor cells. A recent phase I clinical trial investigated the tolerability of cofetuzumab pelidotin in combination with gedatolisib, a pan-class I isoform PI3K/mTOR inhibitor in heavily pretreated metastatic TNBC (NCT03243331). The intervention was generally well tolerated and showed a clinical benefit rate of 27.8% at 18 weeks. The number of patients was too small to detect biomarkers of response; however, the authors speculate that the activity of the regimen could be higher in patients preselected for genomic aberrations in the PI3K pathway [88]. Figure 3 shows Wnt/β-Catenin pathway with crucial targets and the corresponding inhibitors.

### 4.4. JAK/STAT

The JAK/STAT signaling pathway in mammals is composed of four proteins featuring the Janus kinase domain, specifically JAK1, JAK2, JAK3, and TYK2, alongside seven proteins that belong to the signal transducer and activator of transcription (STAT) family [89]. JAK proteins reside in the cytoplasm and are associated with transmembrane receptors. The binding of an extracellular ligand triggers the trans-phosphorylation of JAKs, which in turn phosphorylate STAT monomers. Once activated, STAT proteins translocate to the nucleus, where they play a crucial role in regulating the transcription of various target genes (Figure 3). Dysregulated JAK/STAT signaling was identified as a crucial contributing factor in cancer cell survival and proliferation, tumorigenesis, and immune suppression [89]. Specifically, in TNBC, signal transducer and activator of transcription 3 (STAT3) is highly expressed and associated with tumor initiation, progression, and metastatic spread, as well as angiogenesis, chemoresistance, and immune evasion, making it a target of numerous preclinical and clinical studies [90] (Table 5).

Simvastatin is a widely available lipid-lowering medication that is being investigated for its potential anti-cancer properties. In addition to cellular cholesterol reduction and lipid raft redistribution, simvastatin is able to induce cell cycle arrest by inhibiting the STAT3/SKP2 axis and, through the inhibition of the mevalonate pathway, can also aid in the suppression of the EMT [91,92]. In a phase II study on locally advanced breast cancer, including a TNBC group, simvastatin was administered in conjunction with standard-of-care chemotherapy (NCT04418089). The tumor objective response rate (ORR) and pathological response were higher in patients who received the combined treatment compared to standard-of-care chemotherapy; however, the results were not significant. Nevertheless, the results indicate that the addition of simvastatin may be beneficial [93]. Another trial aims to determine whether simvastatin has a stronger effect on basal TNBC, which, if proven correct, would aid in the selection of patients for treatment (NCT00807950). Currently, a phase II study is recruiting TNBC patients to test if simvastatin in combination with standard-of-care chemotherapy will inhibit EMT and lead to an improved response (NCT05550415).

A promising selective small-molecule kinase inhibitor, tinengotinib, which inhibits the JAK 1/2 and Aurora/STAT3 pathways, was investigated in a recently completed phase I study and was shown to be well tolerated (NCT03654547) [94]. Importantly, it demonstrated preliminary activity in TNBC patients, prompting further study in a phase Ib/II TNBC focused trial (NCT04742959) [94,95]. TTI-101, another small-molecule kinase Inhibitor targeting STAT3, was tested in two phase I trials in advanced solid cancers, including TNBC, as monotherapy (NCT03195699) or in combination with other anti-cancer drugs (NCT05384119).

The recently completed phase I/II clinical trial (NCT03421353) examined AZD9150, a novel antisense nucleotide inhibitor of STAT3, in conjunction with durvalumab and durvalumab plus chemotherapy in patients with advanced solid tumors, including TNBC. Figure 3 shows the JAK/STAT signaling pathway with crucial targets and the corresponding inhibitors.

### 4.5. PI3K/AKT Pathway

Hyperactivation of the PI3K pathway is often observed in TNBC and is linked to aggressive tumor behavior, resistance to chemotherapy, challenging clinical course, and unfavorable outcomes [96,97].

Everolimus is a protein kinase inhibitor of the mTOR signal transduction pathway which has been investigated in numerous clinical trials in TNBC patients as monotherapy and in conjunction with chemotherapy and other targeted agents (NCT01931163, NCT02456857, NCT00827567, NCT02531932, NCT01127763, NCT00930930, NCT01031446). Of note is that two studies evaluated everolimus in the context of TNBC molecular subtypes (Table 6). The FUTURE phase Ib/II (NCT03805399) and FUTURE-SUPER phase II (NCT04395989) clinical trials tested everolimus combined with nab-paclitaxel in metastatic MES tumors with PI3K pathway activation. Both trials demonstrated that the treatment targeted on the basis of molecular subtyping was an encouraging strategy [27,29].

Alpelisib is an oral, alpha-selective PI3K inhibitor that was approved in 2019, in combination with fulvestrant, for the treatment of hormone receptor-positive, HER2-negative, PIK3CA-mutated, advanced, or metastatic breast cancer. Building on this, several trials of alpelisib in TNBC were initiated. Currently, a phase III clinical trial (EPIK-B3, NCT04251533) is investigating alpelisib in combination with nab-paclitaxel versus placebo plus nab-paclitaxel in advanced TNBC with PIK3CA or PTEN aberrations. The same treatment regimen is being tested in a phase II trial (NCT04216472) but in early-stage TNBC with activating mutations in PIK3CA or loss of PTEN. The results of these trials are eagerly awaited.

Ipatasertib and capivasertib, highly specific pan-AKT inhibitors, demonstrated promising results in three randomized phase II trials in TNBC patients, thus spurring further investigations. LOTUS, a phase II trial (NCT02162719), investigated ipatasertib in combination with paclitaxel compared to paclitaxel plus placebo in advanced TNBC [98]. The combined regimen demonstrated longer PFS and OS. The effect was more pronounced in the population with alterations in PIK3CA, AKT1, and PTEN, underscoring the critical role of precise patient selection [98]. Comparable results were obtained in the FAIRLANE phase II trial, but in early TNBC (NCT02301988) [99]. The PAKT trial (NCT02423603), testing capivasertib in combination with paclitaxel, showed similar results to the LOTUS trial [100]. Based on these findings, a phase III trial, IPATunity130 (NCT03337724), was launched comparing ipatasertib and paclitaxel versus placebo and paclitaxel in advanced TNBC with PIK3CA/AKT1/PTEN alterations. However, ipatasertib did not increase efficacy [101]. Mirroring the findings of IPATunity130, the CAPItello-290 phase III trial investigating capivasertib plus paclitaxel in advanced TNBC failed to show improved overall survival compared to paclitaxel plus placebo, even in the cohort with PIK3CA/AKT1/PTEN alterations [28].

**Figure 4:**
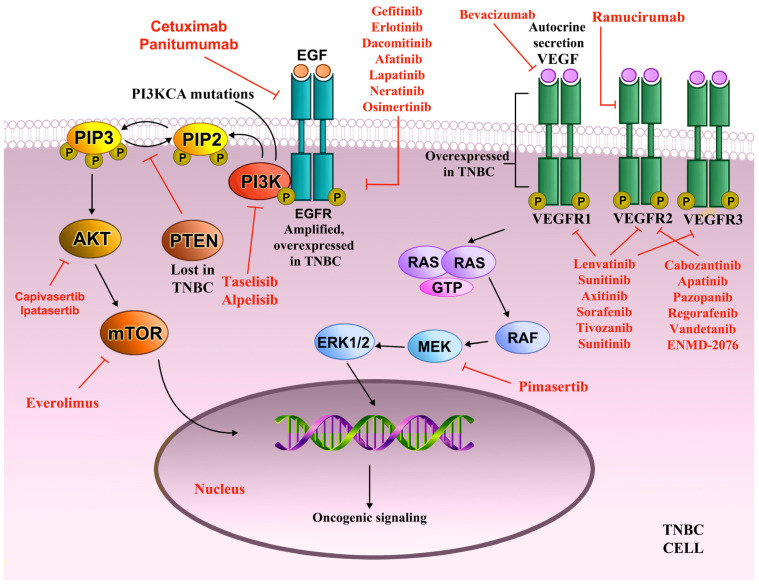
Overview of EGFR, FGFR, PI3K and MEK pathways and their potential inhibitors in triple-negative breast cancer (TNBC). Inhibitors are shown in red. When EGFR and VEGFR are engaged by ligands, they become activated and drive multiple signaling pathways such as PI3K/AKT, RAS/MAPK and others. PI3K transduces growth factor signaling and activates the AKT kinase, which results in the phosphorylation of the mTOR, leading to increased protein synthesis and cell growth. AKT activity is downregulated by the tumor suppressor PTEN. Cetuximab and panitumumab are mAbs directed against the extracellular domain of EGFR. Gefitinib, erlotinib, dacomitinib, afatinib, lapatinib, neratinib, and osimertinib are tyrosine kinase inhibitors that target EGFR and bind to the ATP-binding site of the receptor. Bevacizumab is a mAb against VEGF-A ligand. Ramucirumab is a human IgG1 monoclonal antibody that is directed against the extracellular domain of VEGFR-2. Lenvatinib, sunitinib, axitinib, sorafenib, tivozanib, sunitinib are kinase inhibitors that neutralize VEGFR1-3. Cabozantinib, apatinib, pazopanib, regorafenib, vandetanib and ENMD-2076 are kinase inhibitors that target VEGFR2. Pimasertib is a selective MEK1/2 inhibitor. Everolimus is a protein kinase inhibitor of mTOR. Alpelisib is an oral, alpha-selective PI3K inhibitor. Ipatasertib and capivasertib are highly specific pan-AKT inhibitors. Abbreviations: triple-negative breast cancer (TNBC); epidermal growth factor receptor (EGFR); epidermal growth factor (EGF); vascular endothelial growth factor receptors (VEGFR); vascular endothelial growth factor (VEGF); phosphoinositide 3-kinase (PI3K); mammalian target of rapamycin (mTOR); phosphatidylinositol-3,4,5-trisphosphate (PIP3); guanosine triphosphate (GTP); rapidly accelerated fibrosarcoma (RAF); mitogen-activated extracellular signal-regulated kinase (MEK); extracellular signal-regulated kinase (ERK); monoclonal antibodies (mAbs).

In the preclinical setting, there are strong indications that AKT inhibition may increase checkpoint inhibitor efficacy; therefore, a logical step was to investigate the triple regimen of ipatasertib, paclitaxel, and atezolizumab, a mAb targeting PD-L1 in advanced TNBC. A phase III double-blind, placebo-controlled, randomized trial of triplet regimen ipatasertib/atezolizumab/paclitaxesl IPATunity170 (NCT04177108) was designed with this aim in mind. Unfortunately, this regimen did not achieve better activity compared to anti-PD-(L)1 therapy and chemotherapy alone, leading to early termination of the trial. The PATHFINDER phase II trial evaluated ipatasertib in combination with various non-taxane chemotherapy agents in advanced TNBC (NCT04464174). The ipatasertib plus eribulin was well tolerated and demonstrated encouraging effectiveness independent of PI3K/AKT status, warranting additional investigation [102]. Figure 4 shows the PI3K/AKT signaling pathway with crucial targets and the corresponding inhibitors.

### 4.6. Growth Factor Signaling—EGFR and VEGF

#### 4.6.1. EGFR

High growth factor signaling is a hallmark of MES tumors. Generally, in TNBC, epidermal growth factor receptor (EGFR) is overexpressed in up to 78% of cases and linked with worse clinical course and resistance to chemotherapy [103]. EGFR is engaged by several ligands and, once activated, drives multiple signaling pathways such as PI3K/AKT, RAS/MAPK, STATs, and protein kinase C (PKC) signaling cascade (Figure 4) [104]. EGFR signaling has a key role in the control of crucial cellular processes: proliferation, survival, growth, differentiation, motility, and angiogenesis. In addition, evidence is emerging that EGFR has a number of non-canonical kinase-dependent and kinase-independent roles that promote cancer cell survival [104].

Targeting of receptor tyrosine kinases (RTKs), including EGFR, has two approaches: utilizing mAbs, which either bind to the ligand or extracellular domain of the RTK to block receptor ligand interaction and/or receptor dimerization, and use of small-molecule tyrosine kinase inhibitors (TKIs), which bind to the ATP-binding site of the targeted RTK [105].

Cetuximab and panitumumab are two mAbs directed against EGFR which have previously been approved for the treatment of other types of cancer. Despite the wealth of encouraging preclinical findings [105], cetuximab showed modest results in clinical trials in combination with chemotherapy agents in early and advanced TNBC (NCT00600249, NCT00633464, NCT00463788 [106,107,108]. Panitumumab demonstrated mixed results (Table 7). A phase II clinical trial (NCT00894504) of panitumumab in combination with gemcitabine and carboplatin in metastatic TNBC showed limited efficacy, and in this context, the use of panitumumab alongside chemotherapy was not supported [109]. In contrast, a phase II study (NCT02593175) of panitumumab in conjunction with carboplatin, and paclitaxel as the second phase of neoadjuvant therapy in TNBC patients, which failed the doxorubicin and cyclophosphamide first-line treatment, showed promising results. The trial met its primary endpoint of pathological complete response/residual cancer burden class I of 30.2% compared to 5% in historical controls [110]. Currently, a randomized phase II trial (NCT02876107) is evaluating panitumumab efficacy as a neoadjuvant treatment together with carboplatin and paclitaxel in newly diagnosed inflammatory TNBC.

A number of TKIs targeting EGFR, such as gefitinib, erlotinib, dacomitinib, afatinib, lapatinib, neratinib, and osimertinib, have been approved for use in other malignancies, but none for TNBC [111].

Lapatinib and neratinib are TKIs that target both EGFR and HER2, and as such, they are indicated for the treatment of advanced HER2-positive breast cancers [112]. Lapatinib was the subject of a few clinical trials in TNBC with modest results (NCT01272141, NCT01426880) and is currently being investigated in one study in conjunction with PARP inhibitors (NCT02158507).

Neoadjuvant neratinib, followed by paclitaxel and carboplatin, was tested in a phase II trial in TNBC with enhanced HER2 signaling (NCT03812393). According to the authors, although HER2 is not overexpressed in TNBC, the corresponding signaling pathway may be activated, which can be screened for with CELx assay. However, the screening was unsuccessful, with only 25% of prescreened tumors later being confirmed as HER2-signaling-positive. This led to the early termination of the trial due to a low number of patients with no conclusive results [113]. An ongoing phase I trial is investigating the efficacy and toxicity of neratinib together with ruxolitinib in chemotherapy-pretreated metastatic TNBC (NCT06008275). Neratinib is one of the forty novel drugs that are being evaluated in the I-SPY phase II trial in breast cancer, including a TNBC group (NCT01042379).

Although EGFR overexpression is a marker of TNBC, only a modest benefit was achieved by neutralizing EGFR signaling. EGFR inhibitors have demonstrated great efficacy in tumors dependent on EGFR-activating mutations, such as lung cancer [111]. However, EGFR mutations are rare in TNBC and protein overexpression is mostly due to *EGFR* gene amplification [114]. In addition, the interaction of EGFR with other RTKs broadens the available downstream pathways allowing tumor cells to circumvent EGFR inhibition. Utilizing a range of RTK inhibitors may offer greater clinical benefit in TNBC if toxicity concerns can be resolved. There is evidence that EGFR overexpression is reduced in metastatic TNBC cells compared to the primary tumor, and most trials involved advanced or metastatic TNBC [115]. Therefore, a better selection of patients may be necessary. Figure 4 shows the EGFR pathway with key targets and the corresponding inhibitors.

#### 4.6.2. VEGFR

The vascular endothelial growth factor receptors (VEGFRs) and their corresponding ligands are key drivers of pathological angiogenesis, which provides sufficient nutrients for cells to maintain proliferation, invasiveness, and metastasis in a variety of tumors (Figure 4) [116]. TNBC cells secrete VEGF-A, which correlates with tumor progression and metastasis [117]. Of note is that extensive cross-talk between EGFR and VGFR was observed in TNBC [116]. In addition, it was reported that the activation of the VEGFR pathway has immunosuppressive effects through a wide range of mechanisms [118]. Therefore, anti-VEGF signaling drugs may have synergistic effects with checkpoint inhibitors, providing a rationale for combining these two modalities in clinical trials.

Several agents inhibiting the VEGF/VEGFR pathway are used in cancer treatment, but none have been approved for TNBC. However, there is a wide array of trials that investigated mAbs and TKIs targeting VEGF/VEGFR (Table 8). Bevacizumab is a mAb against VEGF-A that has been thoroughly investigated in TNBC, with almost forty registered studies where bevacizumab was administered as monotherapy or in combination with chemotherapy and other targeted treatments. A phase II trial tested a triple regimen of bevacizumab, atezolizumab, and paclitaxel in patients with advanced TNBC (ATRACTIB, NCT04408118). The safety profile was acceptable and the treatment demonstrated clinical activity [119]. Bevacizumab in combination with gemcitabine and carboplatin displayed improved survival outcomes and manageable safety profile in a phase II study as first-line treatment for metastatic TNBC (NCT01201265) [120]. Ribon 2, a phase III, placebo-controlled trial (NCT00281697) analyzed the activity and toxicity of bevacizumab in conjunction with standard-of-care chemotherapy for the treatment of metastatic, HER2-negative breast cancer. A significant improvement of progression-free survival was observed in the TNBC group [121]. In the neoadjuvant setting, bevacizumab in combination with chemotherapy enhanced the pCR of TNBC patients, as evidenced by the results of phase II (NCT01426880) and phase III (NCT00567554) trials [122,123]. On the other hand, the addition of bevacizumab to adjuvant standard-of-care chemotherapy in early TNBC did not significantly prolong OS in the phase III BEATRICE trial (NCT00528567) [124]. These conflicting results did not diminish the interest in bevacizumab in TNBC treatment, as evidenced by the number of active (NCT03961698, NCT04395989, NCT00861705, NCT05007106) or recruiting trials (NCT05806060, NCT06125080, NCT05192798, NCT04739670, NCT04303988, NCT05749588, NCT06210438, NCT05386524, NCT05180006, NCT05579366).

Lenvatinib is a multiple kinase inhibitor that neutralizes VEGFR1-3. Recently, a phase II trial investigating the efficacy and safety of lenvatinib in combination with pembrolizumab in pretreated advanced solid tumors, including a TNBC cohort, was completed (NCT03797326). The regimen showed clinical activity and a manageable safety profile in the TNBC group; however, there was a single fatality due to a serious treatment-related adverse event [125]. Lenvatinib is undergoing testing in TNBC in several ongoing studies in various settings: in a neoadjuvant setting together with an immune checkpoint inhibitor (NCT04427293); in combination with sindilimab and nab-paclitaxel for the treatment of recurrent and metastatic TNBC (NCT06140576); as monotherapy or in combination with standard-of-care chemotherapy, immune checkpoint inhibitor or targeted therapy in advanced solid tumors including TNBC (NCT05007106). None of the mentioned trials have posted results. Sunitinib is another multiple kinase inhibitor that blocks all VEGFRs. Sunitinib activity was compared to standard-of-care chemotherapy in advanced, pretreated TNBC in a phase II trial (NCT00246571) and was found to be inferior. Sunitinib monotherapy resulted in shorter PFS and OS of patients [126]. Similar results were obtained in a phase III trial (NCT00435409) of sunitinib in combination with capecitabine compared to capecitabine alone in pretreated metastatic breast cancer [127]. Cabozantinib is a multiple kinase inhibitor that targets VEGFR2. Cabozantinib in combination with checkpoint inhibitor nivolumab (NCT03316586) in metastatic TNBC showed limited clinical activity, and the phase II trial did not reach its primary endpoint [128]. On the other hand, a phase I/II trial testing cabozantinib with atezolizumab (NCT03170960) in locally advanced or metastatic solid tumors reported a good toxicity profile and clinical activity of the regimen in several types of malignancies. Unfortunately, results for the TNBC cohort are not yet available [129].

Apatinib is a TKI that selectively inhibits VEGFR2, which has shown clinical activity in both early and advanced settings in TNBC. Apatinib in combination with standard-of-care neoadjuvant chemotherapy in early-stage TNBC demonstrated a good safety profile and high activity (NCT03243838) [130]. These findings led to an increased interest in apatinib for early TNBC, as evidenced by the ongoing trials in this setting (NCT05447702, NCT05556200, NCT05582499). Apatinib was investigated in a phase II trial in combination with anti-PD-1 immune checkpoint inhibitor camrelizumab and eribulin in heavily pretreated advanced TNBC, where it demonstrated encouraging efficacy and manageable safety (NCT04303741) [131]. These findings confirm the results of the earlier phase II study (NCT03394287) of the camrelizumab/apatinib regimen in advanced TNBC, which had a superior clinical response compared to either agent as monotherapy [132]. Furthermore, the addition of apatinib to camrelizumab and a PARP inhibitor fuzuloparib, in patients with recurrent or metastatic TNBC, displayed acceptable toxicity and preliminary antitumor activity (NCT03945604) [133]. A phase II trial (NCT03735082) in locally advanced TNBC suggested that the addition of apatinib to paclitaxel and carboplatin enhanced treatment efficacy. The combined treatment produced a significantly higher pCR rate compared to chemotherapy alone [134]. According to the findings of the NAN trial, administration of apatinib to metastatic, heavily pretreated TNBC patients in conjunction with vinorelbine prolonged progression-free survival with manageable safety [135].

A number of other anti-VEGFR agents are being investigated in TNBC and other subtypes of breast cancer with varying results: ramucirumab, axitinib, pazopanib, regorafenib, sorafenib, tivozanib, vandetanib, sunitinib, and ENMD-2076. Figure 4 shows the VEGFR pathway with crucial targets and the corresponding inhibitors.

To date, the few clinical trials that used subtype-specific therapy focused on the PI3K pathway and VEGFR in MES/MSL tumors [27,29]. Therapeutic strategies should be broadened to include other subtype-specific vulnerabilities in addition to the pathways discussed above. The MES/MSL subtype displays dependencies on EMT genes, genes involved in cellular adhesion and motility. PI3K/AKT/mTOR, JAK/STAT3, TGF-β, Notch and Wnt/β-Catenin pathways are involved in the EMT process in TNBC [136]. In addition, numerous genes connected with EMT are upregulated in TNBC, such as EMT transcription factors (Snail Family Transcriptional Repressor 1, Zinc Finger E-box Binding Homeobox 1), transcriptional regulators (Forkhead Box C2) [136]. On the other hand, proteins associated with the epithelial phenotype often have reduced expression in TNBC. These include E-cadherin, cytokeratins, claudins and occludin, as well as scaffolding proteins [136]. Reversion of EMT is a relatively novel and underused treatment strategy in TNBC that could be especially effective in the MES/MSL subtype. Preclinical research is devising new and better ways to exploit EMT reversal, which will, ideally, culminate in the development of improved agents to examine in clinical trials [137]. Comprehensive analyses of multiomic data have led to the identification of novel therapeutic vulnerabilities that can guide the design of future subtype-specific clinical trials.

## 5. Basal-Like Immune-Activated (BLIA) Tumors

Basal-like tumors are high-histological-grade breast tumors that were originally defined by the upregulation of genes expressed by basal epithelial and normal myoepithelial cells of breast tissue and the expression of high-molecular-weight cytokeratins CK5/6, CK17, CK14, and EGFR [138,139]. Aside from a number of low-frequency mutations, more than 80% of BLIA tumors harbor *TP53* mutations [140]. These tumors are also characterized by the high frequency of HRD-related signature 3, also known as single base substitution signature (SBS3) [9,31]. HRD in breast cancer is associated with germline and somatic mutations in *BRCA1/2* tumor suppressor genes, *BRCA1* promoter methylation, or mutations in other genes involved in homologous recombination such as *RAD51C*, *RAD51D, PALB2*, *BRIP2*, *BARD1*, *ATM*, and *CHEK2* [138,141,142,143]. BLIA tumors possess high chromosomal instability (CIN). In this sense, the *CDK1* gene, although amplified in all TNBC subtypes, displays the highest amplification rate in BLIA tumors [8].

The most distinctive histologic feature of this subtype of breast cancer is the high prevalence of tumor-infiltrating (TIL) and stromal T and B lymphocytes and macrophages [144,145]. Moreover, gene expression analyses showed that BLIA tumors highly express genes involved in immune responses, such as genes regulating T cell function, processing and presentation of antigens, IFN-γ signaling, but also genes encoding immune checkpoint (IC) receptors, which define the exhausted phenotype of immune cells [9]. IC receptors are upregulated as a consequence of immune responses and their primary physiological role is to prevent excessive immune reactions. A number of IC receptors are present on T and NK cells as a result of chronic inflammation in TME: programmed cell death receptor (PD)-1, cytotoxic T lymphocyte antigen (CTLA)-4, T cell immunoglobulin and mucin domain-containing protein 3 (TIM3), lymphocyte activation gene-3 (LAG-3), T cell immunoreceptor with immunoglobulin and ITIM domain (TIGIT) and V-domain Ig-containing suppressor of T cell activation (VISTA) [146,147]. Expression of IC receptors on immune cells and their ligands on tumor cells contributes to immunosuppression and resistance of tumor cells to the antitumor activity of infiltrating lymphocytes in the TME.

Several clinical trials have retrospectively analyzed the responses to IC inhibitors in relation to the TNBC molecular subtypes. In the phase III NeoTRIPaPDL1 trial (NCT02620280), the highest rate of pCR was observed in the BL1 subtype [148]. The findings from the phase III IMpassion 131 trial (NCT03125902) revealed that the pathological complete response rate was enhanced in the atezolizumab plus chemotherapy cohort when compared to the placebo plus chemotherapy cohort, regardless of the PD-L1 status of the metastatic TNBC. This result is inconsistent with the IMpassion 130 study, which found that atezolizumab was more effective in patients with PD-L1-positive TNBC. In the phase III trial IMpassion130 (NCT02425891), which investigated atezolizumab in combination with chemotherapy in patients with metastatic TNB, the BLIA subtype was found to benefit the most [149]. It is possible that patient selection is the underlaying cause of diverging results between these two trials, emphasizing again the significance of comprehensive molecular analyses [150]. In view of these results, the BLIA subtype is likely the most suitable candidate for immunotherapy.

Generally, TNBC has the highest TIL infiltration compared to other molecular subtypes of breast cancer. Although immune cell infiltration has been vastly explored as a prognostic marker in TNBC and has been associated with favorable clinical outcome [151], T lymphocytes in TNBC may exhibit regulatory, exhausted, or a mix of cytotoxic and exhausted phenotypes [152]. Based on the presence of TILs, an enrichment of genes involved in immune regulation in TNBC provides a rationale for immunotherapy with IC inhibitors, therapeutic vaccines, adoptive cell therapy, and cytokines in this subtype of breast cancer.

### 5.1. Immune Checkpoint Inhibitors in Therapy of TNBC

#### 5.1.1. Anti-PD-L1

**Figure 5:**
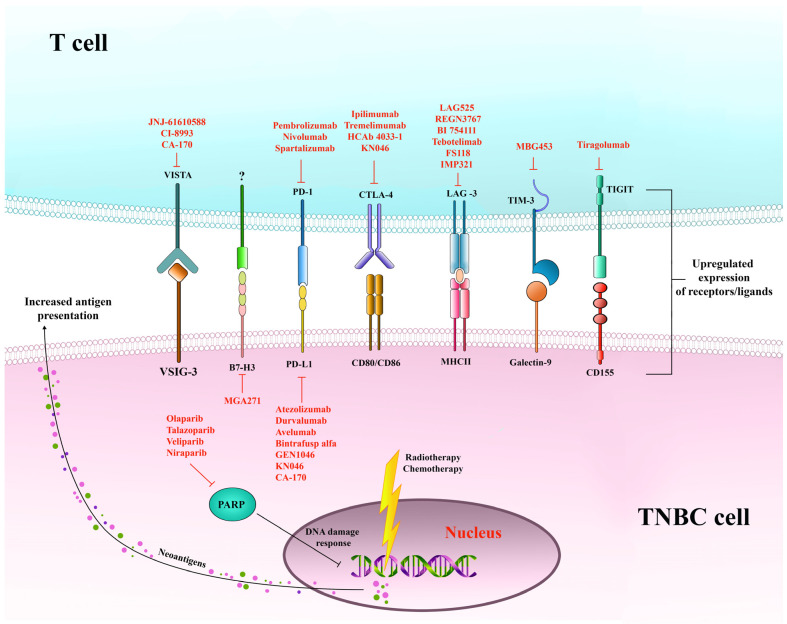
Targets of immunotherapy in triple-negative breast cancer (TNBC). Inhibitors are shown in red. TNBC cells express PD-L1, which interacts with PD-1 receptors on T cells, thereby inhibiting the cytotoxic activity of T cells. The administration of PD-1 or PD-L1 inhibitors disrupts this interaction, allowing T cells to effectively eliminate TNBC cells. Pembrolizumab, nivolumab and spartalizumab are monoclonal antibodies that bind and block PD-1 receptors. Atezolizumab, durvalumab and avelumab are humanized IgG1 monoclonal antibodies that target PD-L1. Bintrafusp alfa is a bifunctional fusion protein composed of the extracellular domain of TGF-β receptor II linked to a human IgG1 monoclonal antibody blocking PD-L1. GEN1046 is a IgG1 bispecific antibody targeting PD-L1 and 4-1BB. KN046 is a bispecific antibody targeting PD-L1 and CTLA-4. CA-170 is a small-molecule inhibitor of PD-L1 and VISTA. Tumor cells produce CD80 and CD86, which engage with CTLA-4 on T cells, resulting in the suppression of T cell-mediated tumor destruction. The use of a CTLA-4 inhibitor can block this interaction, thereby enabling T cells to effectively kill the TNBC cells. Tremelimumab and ipilimumab are monoclonal antibodies that block CTLA-4. HCAb 4003-1 is a human heavy chain-only antibody against CTLA-4. LAG-3 is expressed on T cell membrane and binds to MHC-II on TNBC cells. The interaction with MHC-II obstructs the binding of CD4 and the T-cell receptor and leads to a reduction in T-cell activation. Six LAG-3 antibodies are currently being investigated: five monoclonal antibodies (LAG525, REGN3767, BI 754111, tebotelimab, and FS118) and one LAG-3-Ig fusion protein—IMP321. TIGIT is expressed in T cells and natural killer cells. The presence of CD155 at high levels on tumor cells enables its binding to TIGIT, which in turn induces both direct and indirect downregulation of T-cell responses. Tiragolumab is a human monoclonal antibody targeting TIGIT, demonstrating a synergistic effect when combined with anti-PD-1/PD-L1 therapies. TNBC cells express Galectin 9, which interacts with TIM-3 receptors on T cells, thereby downregulating the activity of T cells. MBG453 is a monoclonal antibody targeting TIM-3. B7-H3 is significantly upregulated in tumor cells as well as in activated immune cells that infiltrate tumors, playing a crucial role in the TNBC evasion of immune response. The receptors for B7-H3 remain unclear. MGA271 (enoblituzumab) is a monoclonal antibody that targets B7-H3. VISTA exhibits dual immunosuppressive functions, acting both as a ligand found on tumor cells and as a receptor expressed on T cells. At present, clinical trials are examining two anti-VISTA antagonistic antibodies (JNJ-61610588, CI-8993) and a small-molecule inhibitor CA-170. PARP enzymes play a crucial role in the repair mechanisms of single-strand breaks in DNA. Inhibition of PARP leads to the formation of double-strand breaks. The accumulation of double-strand breaks triggers apoptotic pathways in the affected cell. This process of immunogenic cell death enhances the release of tumor antigens and promotes localized inflammation. Abbreviations: programmed cell death protein 1 (PD-1); programmed cell death ligand 1 (PD-L1); cytotoxic T lymphocyte antigen (CTLA)-4; lymphocyte activation gene-3 (LAG-3); T cell immunoglobulin and mucin domain-containing protein 3 (TIM-3); T cell immunoreceptor with immunoglobulin and ITIM domain (TIGIT); major histocompatibility complex II (MHCII); V-domain Ig suppressor of T cell activation (VISTA); poly (ADP-ribose) polymerases (PARP); transforming growth factor beta (TGF-β); V-Set and immunoglobulin domain containing 3 (VSIG-3).

During tumorigenesis, oncogenic pathways, genetic and epigenetic factors intrinsic to the tumor itself, as well as extrinsic factors in TME, such as cytokines IFN-γ, interleukin (IL)-6, and tumor necrosis factor (TNF), upregulate the expression of PD-1 ligands (L)1 (B7H1) and L2 (PD-L2) on tumor cells [153,154], leading to cancer immune escape [155]. In TNBC, PD-L1 expression was associated with infiltration of tumor with immune cells, with PD-L1 expression being related mainly to TILs rather than tumor cells [156,157]. Moreover, the expression of PD-L1 and PD-1 in early breast cancer has been associated with higher TIL scores [158]. Altogether, this may be the reason that TNBC seems to represent a good candidate for immune IC therapy with antibodies targeting the PD-1/PD-L1 axis. Figure 5 shows the PD-L1/PD-1 interaction and lists key inhibitors currently in clinical development.

Atezolizumab is a monoclonal IgG1 antibody developed to target PD-L1 and prevent its interactions with PD-1 while allowing interactions with PD-L2, thus reducing autoimmunity. As a single agent, atezolizumab was evaluated in TNBC in a phase I clinical trial (NCT01375842) and was shown to be well tolerated and to provide a durable clinical benefit in patients with metastatic TNBC with stable or responding disease in earlier lines of treatment (Table 9). Although atezolizumab showed some effect, the majority of TNBC patients displayed a poor response to monotherapy [159]. In order to enhance their therapeutic effect, IC inhibitors in clinical trials were often used in combination with chemotherapy, as chemotherapeutic drugs have the ability to induce immunogenic death of tumor cells and increase the release of antigens, activation of dendritic cells (DC)s, induction of tumor-specific cytotoxic T cells and also invasion of lymphocytes in the tumor bed [160].

In this sense, atezolizumab in combination with albumin-bound paclitaxel (nab-paclitaxel) was investigated in the phase III study IMpassion130 (NCT02425891) in unresectable locally advanced or metastatic TNBC. The study showed improved OS and PFS in the treated PD-L1-positive patient population [161], which led to an accelerated approval of this therapy by the FDA for clinical use in patients with tumors expressing PD-L1 > 1% [162]. Unfortunately, in the subsequent confirmatory Impassion131 trial (NCT03125902), atezolizumab in combination with paclitaxel failed to improve PFS or OS, even in the PD-L1+ subgroup [163]. Consequently, FDA issued an alert that protein-bound paclitaxel (abraxane) should not be replaced with paclitaxel in clinical practice. Meanwhile, another phase III clinical trial Impassion03 (NCT0319735) using atezolizumab in combination with neoadjuvant nab-paclitaxel chemotherapy followed by doxorubicin and cyclophosphamide in early-stage TNBC showed pCR, even in the PD-L1-negative cohort [164].

Bifunctional antibodies targeting the immune-related receptors and signaling molecules to harness the immune system are also currently being evaluated in advanced TNBC. One example is bintrafusp alfa, an agent we discussed in Section 4.1 of this review, which enhances immune responses by trapping the immunosuppressive cytokine TGF-β [165]. A clinical trial (NCT04489940) conducted in TNBC identified the high-mobility group AT-Hook 2 (HMG2A) transcriptional regulator, which is upregulated by TGF-β as a potential biomarker in response to bintrafusp alfa [166]. Similarly, IgG1, a bispecific antibody targeting PD-L1 and 4-1BB GEN1046 (DuoBody^®^-PDL1x4-1BB; Genmab), and thus inducing inhibition of an immunosuppressive pathway (PD-L1) and activation of a costimulatory molecule (4-1BB), was developed. GEN1046 was tested in a phase I/II clinical trial (NCT03917381) in the TNBC cohort and showed encouraging preclinical and early clinical activity [167]. Furthermore, KN046, a bispecific antibody targeting PD-L1 and CTLA-4, either as monotherapy or in combination with nab-paclitaxel, has been tested in solid tumors [168].

Aside from chemotherapy, there is a wide range of treatments that use ICI in combination with therapies targeting poly(ADP-ribose) polymerase (PARP), PI3K/AKT, mitogen-activated extracellular signal-regulated kinase (MEK), and VEGF molecular pathways, and radiotherapy, which are presently being investigated in TNBC.

PARP inhibitors exert their antitumor activity by blocking the repair of single-strand DNA breaks and are often used for the treatment of tumors with *BRCA1/2* mutation (Figure 5). As in vitro studies have shown, PARP inhibitors stimulate IFN response and IFN-γ synthesis in tumors with *BRCA* mutation by activating the cyclic GMP-AMP synthase-stimulator of interferon genes (cGAS-STING) and signaling, which further induces PD-L1 expression and recruitment of CD8 T cells [160,169,170]. In this manner, PARP inhibitors increase tumor immunogenicity and susceptibility to PD-L1 inhibition [160]. Clinical trials have further evaluated IC inhibitors in combination with PARP inhibitors. In this sense, PD-L1 inhibitor durvalumab was tested in combination with PARP inhibitor olaparib in a MEDIOLA (NCT02734004) phase I/II clinical study that showed a 50% disease control rate in patients harboring germline *BRCA* mutation [171]. Furthermore, the phase II clinical trial DORA (NCT03167619) evaluated PARP inhibition with or without PD-L1 blockade as chemotherapy-free maintenance therapy for advanced TNBC sensitive to platinum-based chemotherapy. The study reported sustained clinical benefit irrespective of germline *BRCA* mutation or PD-L1 status, which tended to be associated with CR/PR to prior platinum-based chemotherapy, particularly in the arm with PARP inhibitor only [172]. In TNBC harboring *BRCA* mutation, a phase II randomized clinical trial (NCT02849496) is presently evaluating olaparib in combination with atezolizumab.

Among targeted therapies, there is a good preclinical foundation for combining anti-PD-1/PD-L1 with AKT inhibitors. First, the P3K/AKT pathway is activated in approximately 25% of TNBC, which includes the loss of PTEN tumor suppressor, which was, in preclinical tumor models, associated with inhibition of antitumor activity of T cells and resistance to anti-PD-1 therapy [173]. In addition, AKT inhibition was reported to promote the expansion of tumor-specific CD8 T cells with memory-like characteristics [174]. The therapeutic effect of the combination of AKT inhibitor ipatasertib with anti-PD-L1 agent atezolizumab and chemotherapeutics taxanes in patients with advanced TNBC was investigated in a phase Ib clinical trial (NCT03800836), single-arm signal-seeking cohort of the IPATunity130 trial (NCT03337724), and the randomized phase III IPATunity170 trial (NCT04177108). The results of these clinical studies led to the conclusion that in patients with TNBC receiving an ipatasertib/atezolizumab/taxane triplet regimen, molecular characteristics pertaining to *NF1*, *CCND3*, and *PIK3CA* gene alterations and increased immune pathway activity may identify patients with particularly favorable outcome (PFS > 10 months), while *CDKN2A/CDKN2B/MTAP* alterations and lower phosphorylated AKT-S473 levels predicted an unfavorable outcome (PFS < 5 months) [175]. Recently, a phase II clinical trial was initiated examining whether the combination of adjuvant atezolizumab and ipatasertib can reduce the risk of recurrence in patients who have residual disease after treatment with neoadjuvant chemotherapy (NCT04434040). Similarly, another AKT inhibitor, capivasertib, in combination with the PD-L1 inhibitor durvalumab, together with paclitaxel is currently been evaluated in the BEGONIA clinical trial (NCT03742102) [168].

MEK activation has been found in approximately 30% of basal-like breast cancer cases and is often associated with reduced TIL numbers after treatment with neoadjuvant chemotherapy [176]. As was shown in the experimental model of TNBC in mice, MEK1 inhibition upregulated MHC class I and PD-L1 expression, while MEK1 inhibition in combination with PD-1/PD-L1 blockade exerted an enhanced antitumor effect [177]. Similarly, MEK inhibitor cobimetinib in combination with atezolizumab and taxanes as the first-line treatment for advanced TNBC was evaluated in the phase II COLET clinical trial (NCT02322814), which revealed higher ORR and PFS in patients with PD-L1+ tumors [178]. The ongoing InCITe clinical trial (NCT03971409) combining MEK inhibitor binimetinib with the PD-L1 inhibitor avelumab will provide further information on the therapeutic effect of this combination in TNBC.

Hypoxic conditions in solid tumors contribute to a suppressive TME and reduced infiltration of immune cells. Preclinical studies have shown that blockade of VEGFR2 sensitizes breast tumors to PD-1 blockade, as it promotes the secretion of osteopontin by CD8 T cells, which subsequently induces the production of TGF-β by tumor cells, thereby upregulating PD-1 expression in immune cells [179]. The effect of antiangiogenic drugs in combination with IC inhibitors in patients with TNBC is being further tested in clinical trials, as described in chapter 4.6.2.

#### 5.1.2. Anti-PD-1

Pembrolizumab (Keytruda) is a monoclonal antibody that binds PD-1 receptors on T and NK cells and thereby blocks its interaction with PD-L1 and -2. In patients with advanced TNBC, safety and sustained anti-tumor activity of pembrolizumab monotherapy were first shown in 2014 in the KEYNOTE-012 (NCT01848834) phase I clinical study [180,181] (Table 10). The results of the phase II KEYNOTE-086 trial demonstrated the therapeutic efficacy of pembrolizumab monotherapy in previously treated and treatment-naïve metastatic TNBC patients with PD-L1-positive tumors [182]. Furthermore, the phase III clinical trial KEYNOTE-119 (NCT02555657) showed that although OS was similar in the group of patients treated with pembrolizumab and the group of patients treated with chemotherapy, the pembrolizumab treatment effect was more pronounced in PD-L1-enriched tumors [183]. Further investigations have evaluated the therapeutic potential of pembrolizumab in combination with chemotherapy. In the phase III clinical study KEYNOTE-355 (NCT02819518) conducted in patients with locally recurrent unresectable or metastatic TNBC expressing PD-L1 combined positive score (CPS) > 10, pembrolizumab in combination with chemotherapy (nab-paclitaxel; paclitaxel; or gemcitabine plus carboplatin) showed an improvement in OS and PFS of treated patients [184]. This led to its accelerated approval by the FDA in 2020 for the treatment of patients with locally advanced, recurrent, or metastatic TNBC.

Furthermore, in 2021, FDA approved the application of pembrolizumab for the treatment of high-risk and early-stage TNBC in combination with neoadjuvant chemotherapy (carboplatin and paclitaxel followed by doxorubicin or epirubicin and cyclophosphamide) that is continued with pembrolizumab as a single agent after surgery. This approval was based on a phase III KEYNOTE-522 trial (NCT03036488) that revealed improved pCR in patients who were treated neoadjuvantly and adjuvantly with pembrolizumab [185].

As current studies have suggested, the combination of chemotherapy with IC inhibitors significantly improved the antitumor effect of chemotherapy in both neoadjuvant and adjuvant settings. Anti-PD-1 therapy has also been investigated in combination with PARP inhibitors. In this sense, the phase II clinical trial TOPACIO (NCT02657889) reported 47% ORR in TNBC patients with advanced disease with germline BRCA mutations treated with pembrolizumab in combination with nuraparib [186]. Furthermore, there is an ongoing clinical phase II/III study (NCT04191135) evaluating the potential of PARP inhibition to sustain clinical benefit after therapy with chemotherapy and pembrolizumab [187].

Aside from inducing cell death by DNA damage and free radicals, radiotherapy induces immunogenic cell death, increases tumor antigen release, and increases inflammation locally and by provoking abscopal effects. Therefore, the combination of immunotherapy and radiation treatment represents a potential therapeutic strategy in TNBC. Pembrolizumab showed encouraging results and proved safe in combination with radiotherapy in metastatic TNBC patients with poor prognosis who were unselected for PD-L 1 expression [188]. Several ongoing phase II clinical studies are presently evaluating radiotherapy in combination with IC inhibitors and PARP inhibition in metastatic TNBC. In this sense, atezolizumab is evaluated in a clinical trial in combination with radiotherapy and PARP inhibitor talazoparib (NCT03483012) in PDL-1-positive metastatic TNBC and durvalumab in combination with radiotherapy and PARP inhibitor niraparib (NCT04837209) [189].

#### 5.1.3. Anti CTLA-4

CTLA-4 is a transmembrane protein that can be expressed by T cells, B, cells, DCs, stromal and tumor cells. CTLA-4 negatively regulates T cell function by competing with CD28/CD80 costimulatory molecules for the same ligand B7-1 (Figure 5). Due to its higher affinity and avidity for the ligand, CTLA-4 inhibits the role of CD28 as a costimulatory molecule [190,191]. Humanized anti-CTLA-4 antibodies ipilimumab and tremelimumab have shown clinical benefit in some malignancies, including melanoma, renal cell, and lung carcinoma [192]. The effect of this therapy depends on regulatory T cells (Treg), CD8 T cells, and tumor-associated macrophage (TAM) infiltration into tumor tissue [193]. Tregs are induced by IL-2 and play a primary physiological role in the maintenance of self-tolerance and the prevention of autoimmune reactions. However, by producing immunosuppressive cytokines TGF-β and IL-10, Tregs induce immunosuppression in TME [194]. The primary mechanism of action of anti-CTLA-4, aside from blocking the CTLA-4-B7 pathway, is the depletion of Tregs that constitutively express CTLA-4 and inhibit CD4 and CD8 T cells [191,195]. Treg infiltration contributes to the formation of immunosuppressive TME, which plays an important role in the progression and treatment resistance of TNBC [196].

The safety and efficacy of anti-CTLA-4 monotherapy were evaluated for tremelimumab in a phase II clinical trial (NCT02527434) in advanced TNBC, while for ipilimumab, they were evaluated in a phase I study (NCT01502592) in early-stage BC (Table 11). However, these antibodies targeting CTLA-4 have shown suboptimal antitumor activity in BC and severe immunotherapy-related adverse effects (irAEs) that can in great part contribute to lysosomal degradation of CTLA-4 [197]. Efforts have been made in the development of pH-sensitive antibodies that dissociate after lysosomal endocytosis and are recycled to the cell surface and therefore minimize CTLA-4 degradation and circumvent adverse effects [198]. Further efforts were made to create a monoclonal anti-CTLA-4 antibody with the Fc region and improve both its Treg depleting activity and antitumor activity, leading to the development of HCAb 4033-1, which showed a shorter serum half-life, implicating its lower toxicity [199].

Based on the preclinical investigations and due to the poor efficacy of anti-CTLA-4 monotherapy shown in clinical trials, these therapeutics are further tested in combination with other IC inhibitors, chemotherapy, or radiotherapy to improve the treatment outcome of breast cancer patients [197]. In this sense, a small single-arm phase II clinical study (NCT02536794) showed that tremelimumab in combination with PD-L1 inhibitor durvalumab induced some clinical benefit in metastatic TNBC, but the study was terminated due to high risks in the second phase [200]. Another phase II clinical study (NCT02834013) confirmed the significant risks of irAEs of this combination in metaplastic breast cancer [201]. Further studies were directed to lowering the dosage or development of novel anti-CTLA-4 formulations to reduce irAEs [191]. Hence, there are several ongoing studies, including a phase I/II clinical trial (NCT01928394) investigating ipilimumab in combination with nivolumab for advanced or metastatic solid tumors, including TNBC. Simultaneous application of CTLA-4 and PD-1/PD-L1 inhibitors with chemotherapy is currently being evaluated in several clinical studies [197]. Furthermore, a bispecific anti-(PD-L1/CTLA-4) antibody KN046 in combination with nab-paclitaxel was evaluated for the first-line treatment of patients with metastatic TNBC in a multicenter phase II clinical trial (NCT03872791). This study showed survival benefits and tolerable toxicity in the first-line treatment of metastatic patients with TNBC, which was most apparent in PD-L1-positive patients [202]. Aside from chemotherapy, efforts have been made to enhance the therapeutic effect of anti-CTLA-4 therapy in combination with targeted therapy such as MAPK inhibitors, PI3K inhibitors, PARP inhibitors, and radiotherapy. It was reported that the *BRCA1*-mutated TNBCs, due to increased somatic mutational load, show greater numbers of TILs, along with increased expression of immunomodulatory genes, including *PD-1* and *CTLA-4,* when compared to the *BRCA1* wild-type TNBCs [203]. Treatment with cisplatin combined with dual anti-PD-1/CTLA-4 inhibitor substantially augmented antitumor immunity in *BRCA1*-deficient mice as a result of increased infiltration of CD4 and CD8 cells, antitumor cytotoxicity of CD8 T cells, and decreased level of Tregs [203]. Consequently, a phase I clinical trial (NCT02571725) is at present investigating anti-CTLA-4 in combination with PARP inhibitors in *BRCA*-deficient ovarian cancer, showing a favorable therapeutic outcome, indicating the potential of this therapeutic combination in TNBC [204]. Figure 5 shows CTLA-4 interaction with its ligand and lists the main inhibitors.

#### 5.1.4. Next-Generation IC Inhibitor Therapy

Aside from PD-1 and CTLA-4, immune homeostasis is maintained by multiple IC molecules that are present on functionally exhausted immune cells and associated with immunosuppression in cancer. In this sense, expression of LAG-3, TIM-3, TIGIT, VISTA, or B7/H3 IC molecules on immune cells may contribute to resistance to anti-PD-1 or anti-CTLA-4 treatment [152]. To date, four types of IC inhibitors, anti-PD-1, PD-L1, CTLA-4, and LAG-3 mAbs, have been approved by FDA for therapy in oncology. In recent years, therapies blocking other IC pathways have been investigated in malignancies, including TNBC [205].

The LAG-3 molecule is expressed mostly by activated T cells, but it can also be found on NK, B, and dendritic cells. Although the mechanism of action of LAG-3 is not completely understood, LAG-3 interacts with MHC class II molecules and inhibits cytokine production by T cells and T cell expansion (Figure 5) [206]. Six LAG-3 antibodies are currently being investigated: five monoclonal antibodies (LAG525, REGN3767, BI 754111, tebotelimab, and FS118) and one LAG-3-Ig fusion protein (IMP321) (Table 11). LAG-3 is often co-expressed with PD-L1 and upregulated on TILs in TNBC, indicating that TNBC is likely a suitable candidate for cotreatment with LAG-3 and PD-1/PD-L1 inhibitors [207]. However, the coexpression of these two ICs did not confer additional survival benefits in TNBC patients [208]. Although dual blockade of LAG-3 and PD-1 showed synergy in preclinical models, LAG525 antibody in combination with carboplatin or anti-PD-1 antibody (spartalizumab) or as a triplet therapy after a phase II clinical study (NCT03499899) in patients with advanced TNBC did not meet preliminary efficacy criteria [206]. Another phase II clinical trial (NCT02460224) investigated LAG525 in combination with PD-L1 inhibition in advanced solid malignancies and showed modest antitumor activity. However, the patients responding to the therapy showed a tendency towards having higher levels of immune gene expression, including *CD8* and *LAG3*, in tumor tissue at baseline [209].

TIM-3 negatively regulates T cell functions and is also expressed on NK cells and macrophages. TIM-3 induces the expansion of immunosuppressive myeloid-derived suppressor cells (MDSCs). Its ligands, phosphatidylserine, galectin-9, and cancer-embryonic antigen cell adhesion molecul1 (CEACAM)-1, by binding to TIM-3, induce T cell death by apoptosis (Figure 5) [210]. In early breast cancer, TIM-3 expression was correlated with improved breast cancer survival [211]. These findings were also supported in a study on TNBC, indicating TIM-3 as an independent positive prognostic factor in TNBC, despite its association with poor clinical and pathologic features [212]. Upregulation of TIM-3 on TILs has been related to adaptive resistance to immunotherapy with anti-PD-1 or anti-CTLA-4 agents in some tumors [213]. Currently, one anti-TIM-3 mAb (MBG453) is being investigated in a phase I/II clinical trial in patients with advanced malignancies (NCT02608268). No clinical results have been reported yet (Table 11).

TIGIT is expressed on cytotoxic and helper T cells, Tregs cells, and NK cells, while its main ligand CD155 (PVR) is expressed on MDSCs and cancer cells (Figure 5) [214]. TIGIT blockade enhances antitumor effector T-cell and NK cell responses and reduces the suppressive capacity of Tregs [215]. In TNBC, the expression of TIGIT was shown on immune cells in TME and CD155 on tumor cells concomitantly with PD-1 and PD-L1 expression on immune and tumor cells, respectively [216], thus indicating the potential of therapeutic targeting of TIGIT in TNBC. However, the sole blockade of TIGIT showed limited antitumor efficacy, while the blockade of TIGIT in combination with anti-PD-1/PD-L1 therapy showed enhanced antitumor effect in mice even in anti-PD-1-resistant tumor models [205,207]. Recent clinical trials on TIGIT blockade in cancer have been initiated predominantly in combination treatments. In TNBC, TIGIT inhibitor tiragolumab in combination with atezolizumab and chemotherapy was investigated in a phase Ib clinical trial and showed encouraging results (NCT04584112) [215] (Table 11).

B7-H3 is a member of the B7 family of IC molecules. This molecule is highly expressed on cancer cells and activated tumor-infiltrating immune cells and contributes to tumor immune evasion (Figure 5). In breast cancer, B7-H3 expression was correlated with greater tumor size and lymphatic invasion [217]. Moreover, in patients with TNBC, B7-H3 was reported to be specifically enriched in TAMs containing mostly M2 macrophages that produce cytokines TGF-β, IL-4, IL-13, IL-10, and macrophage colony-stimulating factor and contribute to immunosuppression in TME. Moreover, B7-H3 strongly correlated with poor clinical prognosis in TNBC, as TAMs expressing high levels of B7-H3 may facilitate tumor cell dissemination by inducing reconstruction of the extracellular matrix, angiogenesis, and dampening T cell infiltration into tumor tissue [218]. In addition, patients with TNBC expressing high levels of B7-H3 and low levels of PD-L1 showed immuno-cold features such as low immune cell infiltration, high collagen level, and low therapeutic responses to IC inhibition. Targeting B7-H3 by blocking antibody significantly suppressed TNBC growth in a mouse model, increased immunogenicity of the tumor and response to anti-PD-1 immunotherapy [219]. In summary, the combination of IC inhibitors targeting B7-H3 and PD-1/PD-L1 is currently being explored in clinical trials in advanced malignancies (Table 11).

VISTA is an inhibitory IC molecule expressed on T cells, mostly on naïve CD4 and Tregs cells, and on antigen presenting cells (Figure 5). In TNBC, the expression of VISTA was reported to be gradually increased with the degree of stromal TIL infiltration. The high expression of VISTA was strongly linked to the higher proportion of CD8 T cells and immunostimulatory subset M1 of macrophages, thus associating this molecule with favorable prognosis in these patients [220]. Ongoing clinical trials are presently investigating two anti-VISTA antagonistic antibodies and one small molecule with both VISTA and PD-L1 in phase I clinical trials (NCT02671955, NCT04475523, and NCT02812875, respectively) and so far have shown a growth-inhibitory effect in refractory solid tumors (Table 11) [221]. Figure 5 shows LAG-3, TIM-3, TIGIT, VISTA interactions with their ligands and lists the main inhibitors.

Furthermore, humanized agonistic antibodies against costimulatory molecules such as tumor necrosis factor receptor TNFR superfamily members 4-1BB, OX40, CD40, and glucocorticoid-induced TNFR-related receptor (GITR) are currently being investigated in preclinical and clinical settings. Clinical evaluations of these therapeutics are mostly conducted in combination with PD-1/PD-L1 blockade in phase I/II clinical trials in advanced solid tumors, including TNBC [168,207].

### 5.2. Immunotherapies Modulating TME

Several therapeutic strategies are directed at modulating TME by targeting immunosuppressive cells, soluble metabolites, and enzymes, and the application of cytokines in order to increase tumor immunogenicity, facilitate the antitumor activity of immune cells, and potentiate therapeutic efficacy of other immunotherapeutic agents.

Infiltration of TAMs into tumor tissue is associated with worse disease prognosis in breast cancer [222,223]. Targeting TAMs through the blockade of colony-stimulating factor 1 receptor (CSF-1R), which is a key regulator of TAM differentiation and survival, by monoclonal antibodies showed an antitumor effect in animal models. This effect was induced by depleting TAMs while increasing CD8/CD4 T cell ratio [222]. Agents targeting CSF-1R are investigated in phase I/II clinical trials in TNBC mostly in combination with chemotherapy and anti-PD-1/PD-L1 agents (NCT04331067) (Table 12) [168].

Adenosine signaling is an important immunosuppressive pathway within the TME. While ATP activates innate immune responses through danger-associated molecular pattern receptors, adenosine suppresses immune responses by binding to its A2a receptor and prevents excessive tissue damage. Soluble adenosine is produced by dephosphorylation of extracellular ATP by ectonucleotidase CD73, which is highly expressed in the TME and upregulated due to hypoxic conditions, and by adenosine itself in a positive feedback mechanism [224]. Several currently ongoing phase I clinical trials are testing monoclonal antibodies and small-molecule inhibitors directed against CD73 both as monotherapy and in combination with PD-1/PD-L1 inhibitors in advanced solid tumors [225]. In patients with advanced TNBC, a phase II clinical study (NCT03207867) is examining the A2a adenosine receptor inhibitor NIR178 in combination with the anti-PD-1 antibody, while the BEGONIA phase Ib/II trial (NCT03742102) is investigating CD73 inhibitor oleclumab in combination with anti-PD-L1 antibody, durvaluamb, and paclitaxel [168]. A dual antagonistic antibody developed against A2a and A2b adenosine receptors in combination with PD-1 inhibitor showed good tolerability and objective response in a phase I clinical study (NCT0369756) in patients with advanced malignancies, including TNBC, which supported additional phase II combination studies [226].

Cytokines mediate cell-to-cell signaling, regulate cell differentiation and immune responses, and therefore represent important candidates for cancer immunotherapy. IL-2 and IFN-α are the only immunostimulatory cytokines that are approved by the FDA for cancer treatment, but their use is limited due to the high toxicity [227]. IL-12 is one of the cytokines with antitumor activity that is currently investigated in oncology, mostly for intratumoral application. In this sense, IL-12 in the phase I clinical study in advanced refractory TNBC increased the density of CD8 TILs, while the phase II KEYNOTE-890 trial (NCT03567720) investigating the intratumoral application of the plasmid encoding IL-12 by electroporation and pembrolizumab in metastatic TNBC patients showed an increase in objective responses [228,229]. Furthermore, NKTR-214, an engineered cytokine that specifically stimulates IL-2 receptor, was tested in a phase I trial (NCT02869295) in metastatic solid tumors, including TNBC. The study showed a favorable safety profile and a substantial increase in CD8 T and NK cells in TME. This led to further investigations of NKTR-214 in combination with PD-1 inhibitor in a phase I/II trial (NCT02983045) [230]. Aside from cytokines that stimulate antitumor immune responses, the strategy involving the blockade of tumor-promoting and immunosuppressive cytokine signaling is also being investigated. In this sense, the inhibitory antibody against IL-6 receptor is currently being investigated in oncology in a phase II trial for patients with advanced TNBC in combination with anti-PD-1 therapy and nab-paclitaxel in a phase II clinical study (NCT03424005) [168].

Inhibition of enzymes that induce differentiation and activation of suppressive immune cells and inhibiting antitumor activity of T cells is another strategy in targeting immunosuppression in TME. In this sense, one of the potential targets is indoleamine 2,3-dioxygenase (IDO), a tryptophane degrading enzyme that promotes differentiation of Tregs and decreases the amount and activity of CD8 T cells [194,231]. As TNBC showed higher levels of PD-L1 and IDO compared with other breast cancer subtypes, IDO inhibitors are being evaluated in combination with PD-1/PD-L1 inhibition in preclinical and clinical settings [207,232]. Another potential therapeutic target is an enzyme, arginase, produced by MDSC, suppressive M2 macrophages, and tumor cells induce immunosuppression by depleting TME from T cells. The therapeutic activity of selective arginase inhibitors is being investigated in patients with advanced malignancies in phase I/II clinical trials, usually in combination with PD-1/PD-L1 inhibitors [207].

Another immunomodulating therapeutic strategy involves targeting toll-like receptors (TLRs) with agonistic antibodies. Stimulation of TLRs leads to activation of innate immune cells (DCs and macrophages) and enhanced antigen presentation. Current clinical trials are mostly investigating TLR agonists in combination with anti-PD-1/PD-L1 IC inhibitors. CMP-001 is a virus-like particle containing a TLR9 agonist that induces synthesis of IFN-α by plasmacytoid DCs and transcription of IFN-inducible genes in T and NK cells. Intratumoral application of CMP-001 in combination with IC inhibitors is currently being investigated in a phase II trial (NCT04916002) in patients with metastatic TNBC [233]. The agonist of TLR9, SD-101, is a synthetic CpG oligonucleotide and was investigated in neoadjuvant therapy in combination with pembrolizumab in patients with breast cancer (NCT01042379; I-SPY). A dual TLR agonist targeting TLR7 and TLR8 which is delivered intratumorally in combination with OEGylated IL-2 (NKTR-214) was investigated in a phase I trial. The effect of this therapy was further investigated in patients who progressed after therapy with IC inhibitors in the phase II REVEAl trial and in patients naïve to therapy with IC inhibitors in the phase I/II trial (NCT03435640), comprising also the IC inhibitor nivolumab [168].

In summary, it has been well established that due to the TIL infiltration, expression of immune-related genes and signal transduction pathways, BLIA represents a subtype of TNBC which is more likely to benefit from treatments with IC inhibitors. In this sense, better clinical results have been achieved in the combination of IC inhibitors with chemotherapy, PARP inhibitors and radiotherapy, which increase immunogenicity of the treated tumor. Furthermore, immune profiling and longitudinal monitoring of metastases during the course of therapy may give more insight and aid clinicians in decision making and selection of IC inhibitors. In the light of this, recent advances in clinical and preclinical research have introduced novel IC inhibitors and other agents that target immunosuppression or induce immune activity in TME that still need to be better evaluated in clinical settings.

## 6. BLIS Characteristics and Implications for Therapy

BLIS is the most frequent of the TNBC subtypes [9], with the worst prognosis and the highest recurrence rate [144]. Unlike BLIA, this basal-like subset lacks immune activation and expression of immune-related genes, and accordingly is unresponsive to immunotherapy with IC inhibitors [9]. Moreover, the BLIS subtype has been shown to highly express the *VTCN1* gene encoding the B7-H4 IC receptor that negatively regulates T cell activation [8,234]. BLIS tumors are characterized by upregulation of the cell cycle, activation of DNA repair mechanisms, and heightened genomic instability, thus exhibiting highly proliferative properties. In this sense, this TNBC subtype shows increased expression of proliferation-related genes, including *CENPF*, *BUB1*, *PRC1*, and *SOX* family transcription factors [8].

Similarly to BLIA, the BLIS subtype displays chromosomal instability scores, enrichment for DNA damage repair pathways, *TP 53* loss and a high overall number of low-frequency mutations [7]. More importantly, BLIS constitutes 65% of breast cancer with HRD mutations. It has been shown in clinical practice that adding carboplatin to standard neoadjuvant chemotherapy significantly increased pCR in TNBC compared to HER2+ breast cancer [122]. HRD score, a copy-number-based biomarker [235], was proposed as a biomarker (not restrictive to *BRCA1/2* germline mutational status) for the characterization of patients who may benefit from DNA-damaging agents. In this sense, high-HRD tumors were shown to derive substantial benefit from platinum-based compounds, as platinum salts have been shown to induce DNA crosslinks and strand breaks more effectively in tumors that are inherently defective for DNA repair mechanisms, such as HRD and *BRCA1/2* mutations [236]. Furthermore, BLIS tumors with a low HRD score were associated with worse disease prognosis, while tumors with a high HRD score correlated with better disease prognosis [9]. RAD51 is a critical effector of homologous recombination involved in DNA repair mechanisms for double-strand breaks. BRCA-deficient tumors show reduced or obliterated RAD51 foci formation and increased sensitivity to platinum salts [237]. Furthermore, the low level of RAD51 during the S/G2 phase of the cell cycle was reported to be in 87% concordance with HRD status and to independently predict pCR following platinum-based neoadjuvant chemotherapy in unselected TNBC and may aid in the selection of patients sensitive to platinum-based chemotherapy [238].

According to the classification of TNBC based on copy number alteration (CNA) data proposed by one study [9], six clusters were identified based on CNA peaks. Based on this, the high-HRD BLIS showed *Chr9p23/Chr13q34* gene segment amplification, while the low-HRD BLIS showed the prevalence of *Chr12p13* amplification, *Chr20q13* amplification, and *Chr8p21* deletion CNA subtypes [9]. Thus, the unfavorable prognosis shown in patients with low-HRD BLIS tumors can be in part attributed to its inclination towards whole-genome doubling. These findings may direct future investigations to focus on CNA peaks in the light of potential therapeutic targeting [239].

According to one study on metabolomic features of TNBC which was based mostly on dependency on lipid metabolism and glycolysis, BLIS tumors were characterized as a subtype with the upregulation of metabolites related to oxidation reaction and glycosyl transfer, and the ones having the lowest level of metabolic dysregulation. The study defined N-acetyl-aspartyl-glutamate as a crucial tumor-promoting metabolite and a potential therapeutic target for high-risk BLIS tumors [19]. Due to the aberrant DNA damage repair pathways inherent in the BLIS subtype, these tumors exhibit a heightened sensitivity to PARP inhibitors.

### PARP Inhibition

Characteristics such as upregulation of cell cycle processes, genomic instability due to HRD, activation of DNA repair mechanisms, and downregulation of immune response genes define BLIS tumors as candidates for therapy with PARP inhibitors and chemotherapy.

Approximately 10–20% of patients diagnosed with TNBC possess germline mutations in the *BRCA1/2* tumor suppressor genes responsible for the repair of double-strand DNA breaks. Cells lacking functional *BRCA* genes show less accurate repair mechanisms, resulting in more genomic instability and an increased risk of developing certain types of cancers [240]. PARP enzymes assist in the repair of single-strand breaks through base excision repair. Inhibition of PARP results in the trapping of the PARP-DNA complex at the replication fork, causing a transition from single- to double-strand breaks (Figur 5). The accumulation of double-strand breaks subsequently leads to apoptosis of the affected cell. In patients with breast and ovarian cancer harboring deleterious *BRCA* mutations, PARP inhibitors increased PFS. In this sense, PARP inhibitor olaparib was initially approved for the treatment of advanced ovarian cancer associated with germline BRCA mutations. A few years later, in 2018, olaparib became the first PARP inhibitor approved by the FDA for the second-line treatment of breast cancer patients with *BRCA1/2* germline mutations who have previously undergone chemotherapy or hormone therapy if hormone receptor-positive [241]. The phase III randomized multicenter olympiA trial (NCT02032823) demonstrated clinical benefit in patients with high-risk TNBC and BRCA1/2 germline mutation who have completed surgery and/or chemotherapy and were afterward treated with adjuvant PARP inhibitor olaparib [242,243]. Similar findings were obtained for PARP inhibitors talazoparib and veliparib in EMBRACA (NCT01945775) and BROCADE 3 (NCT02163694) phase III clinical trials, respectively [244,245]. PARP inhibitors were also used in clinical settings in combination with chemotherapy. Additionally, the I-SPY 2(NCT01042379) phase II multicenter clinical trial reported higher estimated rates of pCR in the TNBC population treated with another PARP inhibitor, veliparib, in combination with carboplatin [246]. Similarly, the BROCADE3 phase III clinical trial compared veliparib versus placebo in combination with carboplatin and paclitaxel, and continued as monotherapy if carboplatin and paclitaxel were discontinued before progression, in patients with HER2-negative advanced breast cancer and germline *BRCA1* or *BRCA2* mutation. According to this trial, the addition of veliparib to a highly active platinum doublet, with continuation as monotherapy if the doublet were discontinued, resulted in significant and durable improvement in PFS in patients [245].

Aside from *BRCA1/2* mutational status, the high HRD level may suggest sensitivity of the TNBC subtype to PARP inhibitors, as was shown in the phase II RIO clinical trial (EudraCT 2014-003319-12). Furthermore, reduced or obliterated RAD51 foci formation was associated with increased sensitivity to PARP inhibitors [247].

Resistance to PARP inhibitors, aside from the increase in drug efflux, can be induced by various mechanisms, such as genomic reversal of *BRCA1/2* mutations, increased HR repair capacity, restoration of replication fork protection, and other mechanisms, such as STAT3, clonal selection, hypoxia, etc. [248].

Further investigations were aimed at overcoming resistance to PARP inhibition. In this sense, it has been shown that simultaneous application of low doses of DNA methyltransferase inhibitors with PARP inhibitors led to the increased efficacy of PARP inhibitors [239,249]. Moreover, as PI3K is crucial in maintaining DNA structure stability and facilitating DNA repair, the PI3K inhibitors lead to DNA damage, downregulate the expression of BRCA1/2, and enhance the sensitivity of BRCA-proficient TNBC to PARP inhibition [250].

Figure 5 is an overview of receptors which are major therapeutic targets in immunotherapy of TNBC.

Recently, according to an integrative analysis combining somatic mutation, copy number aberrations (CNAs) and gene expression profiles, TNBC subtype-based therapy-oriented clinical trials have been conducted. In this sense, a phase 2 clinical study (NCT04395989) is currently evaluating the efficacy and safety of first-line treatments of untreated metastatic or recurrent TNBC according to the molecular subtype [27]. In this trial, based on the VEGF gene signature of BLIS subtype [10], bevacizumab was evaluated in as the first-line treatment of BLIS molecular subtype. The findings of this trial showed the potential clinical benefits of molecular subtype-based treatment of TNBC, suggesting further clinical investigation of this approach in phase 3 randomized clinical trials further assessing the efficacy of subtyping-based therapy regimens [27].

## 7. Conclusions

TNBC is the most aggressive and lethal type of breast cancer. In recent years, great strides have been made in advancing TNBC treatment beyond chemotherapy; however, clinical management remains challenging. As has been highlighted in the present review, numerous targeted agents are in active development for the treatment of TNBC but have generated mixed results in clinical trials. We believe that the chief underlying factor for inconsistent results may be inadequate patient selection. TNBC is an extremely heterogeneous disease that can be subdivided into several molecular subtypes with distinct clinical behaviors and sensitivity to targeted treatment. TNBC heterogeneity is yet to be fully comprehended. Going forward, it will be imperative to stratify patients according to the molecular subtypes in order to provide more precise treatment and enhance the efficacy of therapeutics, therefore deriving the most benefit for the patients. To this end, two pivotal milestones need to be achieved. First, molecular subtyping of TNBC needs to be standardized. Currently, there are several competing models. Secondly, despite technological advancements, determining gene expression profiles, which are needed for TNBC subtyping, is cumbersome and expensive, which makes it not practical to use in the everyday clinic. Similarly to how ER, PR, and HER2 expressions are used to define TNBC, a surrogate IHC method to determine molecular subtypes of TNBC needs to be devised.

## Figures and Tables

**Table 1:** Summary of clinical trials targeting the androgen receptor in TNBC.

NCT Number/Phase	AR-Directed Agent	AR Positivity by IHC	Result	Mechanism	Status
NCT00468715 Phase II	Bicalutamide	10%	19% Clinical benefit rate	AR antagonist	Completed
NCT02348281 Phase II	Bicalutamide	10%	Terminated without results	AR antagonist	Terminated
NCT03055312 Phase III	Bicalutamide	10%	Terminated without results	AR antagonist	Terminated
NCT01889238 Phase II	Enzalutamide	10%	25–33% Clinical benefit rate	AR antagonist	Completed
NCT02750358 Phase II	Enzalutamide	1%	Well Tolerated	AR antagonist	Active, not recruiting
NCT02689427 Phase II	Enzalutamide	10%	42% Response rate	AR antagonist	Completed
NCT06099769 Phase II	Enzalutamide	10%	Recruiting, no results posted	AR antagonist	Recruiting
NCT04103853 Phase I	Proxalutamide	1–25% (low), 26–75% (moderate), and >75% (high)	38.5%. Disease control rate	AR antagonist	Completed
NCT03383679 Phase II	Darolutamide	10%	24.5% Clinical benefit rate	AR antagonist	Completed
NCT02368691 Phase II	Enobosarm	10%	Terminated due to lack of efficacy	Selective AR modulator (SARM)	Terminated
NCT02971761 Phase II	Enobosarm	50%	25% Clinical benefit rate	Selective AR modulator (SARM)	Completed
NCT02580448 Phase I/II	Seviteronel	10%	33% Clinical benefit rate	Androgen synthesis inhibitor	Completed
NCT04947189 Phase I/II	Seviteronel	10%	Recruiting, no results posted	Androgen synthesis inhibitor	Recruiting
NCT01990209 Phase II	Orteronel	10%	Failed to meet the primary end point of improved OS	Androgen synthesis inhibitor	Completed
NCT02457910 Phase I/II	Enzalutamide	10%	35.7% Clinical benefit rate for all TNBC, 75% for LAR	AR antagonist	Terminated
NCT03207529 Phase I	Enzalutamide	1%	No results posted	AR antagonist	Completed
NCT02605486 Phase I/II	Enzalutamide	1%	33% Clinical benefit rate	AR antagonist	Active, not recruiting
NCT06365788 Phase II	Bicalutamide	1%	Recruiting, no results posted	AR antagonist	Recruiting
NCT05095207 Phase I/II	Bicalutamide	1%	Recruiting, no results posted	AR antagonist	Recruiting
NCT03090165 Phase I/II	Bicalutamide	10%	Recruiting, no results posted	AR antagonist	Recruiting

Abbreviations: androgen receptor (AR).

**Table 2:** Summary of clinical trials targeting the TGF-β pathway in TNBC.

NCT Number/Phase	Agent	Group of Patients	Result	Status
TGF-β Pathway				
NCT01401062 Phase II	Fresolimumab + Radiotherapy	Metastatic breast cancer	Intervention was well tolerated, higher fresolimumab dose had a good systemic immune response and longer median OS	Completed
NCT03192345 Phase I	SAR439459	Advanced solid tumors	Discontinued due to the questionable efficacy and high bleeding risk	Terminated
NCT04291079 DRAGON Phase I	SRK-181 + anti-PD-(L)1 antibody therapy	Locally Advanced or Metastatic Solid Tumors	Well tolerated as monotherapy and in combination with anti-PD-(L)1.	Active, not recruiting
NCT02947165 Phase I	NIS793 + Spartalizumab	Advanced Malignancies	Manageable safety, and early clinical activity	Completed
NCT01646203 Phase I	LY3022859	Advanced Solid Tumors	Unsafe due to uncontrolled cytokine release	Completed
NCT02725489 Phase II	Vigi + Durvalumab	Advanced BRCA-wt TNBC	Well tolerated with encouraging preliminary efficacy	Completed
NCT02672475 Phase I	Galunisertib + Paclitaxel	Metastatic TNBC	No result available	Completed
NCT04489940 Phase II	Bintrafusp alfa	Metastatic TNBC	Probability of success too low to justify study continuation	Terminated
NCT04296942 BrEAsT Phase I	Bintrafusp alfa + BN-brachyury recombinant vector-based cancer vaccine	Advanced Breast Cancer	Slow accrual	Terminated
NCT04789668 Phase I/II	Bintrafusp alfa + pimasertib	TNBC with brain metastasis	No result available	Completed
NCT03579472 Phase I	Bintrafusp alfa + Eribulin Mesylate	Metastatic TNBC	No result available, development discontinued	Terminated

Abbreviations: triple-negative breast cancer (TNBC); programmed cell death ligand 1 (PD-L1).

**Table 3:** Summary of clinical trials targeting the Notch pathway in TNBC.

NCT Number/Phase	Agent	Group of Patients	Result	Status
Notch Pathway				
NCT00106145 Phase I	MK-0752	Advanced Breast Cancer	Manageable safety, and early clinical activity	Completed
NCT00645333 Phase I/II	MK-0752 + Docetaxel	Metastatic Breast Cancer	Manageable safety and encouraging preliminary efficacy	Completed
NCT01151449 Phase II	RO4929097	Advanced, Metastatic, or Recurrent TNBC	Manageable toxicity, evidence of preliminary activity	Terminated
NCT01238133 Phase I	RO4929097 + Paclitaxel + Carboplatin	Stage II or Stage III TNBC	Well tolerated	Terminated
NCT01071564 Phase I	RO4929097 + Vismodegib	Metastatic breast cancer	No result available	Terminated
NCT01876251 Phase I	PF-03084014 + Docetaxel	Advanced breast cancer including TNBC	Well tolerated and demonstrated clinical efficacy, terminated due to change in strategy of PF-03084014 development.	Terminated
NCT02299635 Phase II	PF-03084014	Advanced TNBC	Terminated due to change in strategy of PF-03084014 development.	Terminated
NCT02338531 Phase II	PF-03084014	Chemoresistant TNBC	Terminated due to the discontinuation of the development of PF-03084014-04	Withdrawn
NCT02784795 Phase I	LY3039478 + Taladegib + Abemaciclib + Cisplatin + Gemcitabine + Carboplatin + LY3023414	Advanced or Metastatic Solid Tumors including breast cancer	Low efficacy and high toxicity	Completed
NCT04461600 Phase II	AL101	Recurrent or advanced Notch activated TNBC	Terminated by sponsor’s decision	Terminated
NCT03000257 Phase I	Rovalpituzumab tesirine + Venetoclax + Budigalimab	Advanced solid tumors, including breast cancer	Acceptable levels of toxicity and promising efficacy	Completed

Abbreviations: triple-negative breast cancer (TNBC).

**Table 4:** Summary of clinical trials targeting the Wnt/β-Catenin pathway in TNBC.

NCT Number/Phase	Agent	Group of Patients	Result	Status
Wnt/β-Catenin Pathway				
NCT01351103 Phase I	LGK974 + Spartalizumab	Advanced solid tumors including TNBC	Manageable toxicity and limited activity	Completed
NCT01302405 Phase I	PRI-724	Advanced solid tumors including breast cancer	Acceptable safety profile, terminated due to slow accrual	Terminated
NCT02020291 Phase I	Foxy-5	Metastatic breast cancer	Low toxicity	Completed
NCT02655952 Phase I	Foxy-5	Metastatic breast cancer	Low toxicity	Completed
NCT03883802 Phase II	Foxy-5 + FOLFOX regimen	Wnt-5a low colon cancer	No result available	Active, not recruiting
NCT01973309 Phase I	Vantictumab + Paclitaxel	Metastatic breast cancer including TNBC	Manageable safety and encouraging preliminary activity	Completed
NCT02776917 Phase I	Zilovertamab + Paclitaxel	Advanced HER2-negative breast cancer	Manageable safety, and early clinical activity	Completed
NCT03243331 Phase I	Cofetuzumab pelidotin + Gedatolisib	Metastatic TNBC	The intervention was generally well tolerated and showed promising clinical activity.	Completed

Abbreviations: triple-negative breast cancer (TNBC); folinic acid, fluorouracil, and oxaliplatin chemotherapy regimen (FOLFOX); human epidermal growth factor 2 (HER2).

**Table 5:** Summary of clinical trials targeting the JAK/STAT pathway in TNBC.

NCT Number/Phase	Agent	Group of Patients	Result	Status
JAK/STAT				
NCT04418089 Phase II	Simvastatin + neoadjuvant chemotherapy	Locally advanced breast cancer	Well tolerated and demonstrated clinical efficacy	Completed
NCT05550415 Phase II	Simvastatin + neoadjuvant chemotherapy	Advanced TNBC	No result available	Recruiting
NCT03654547 Phase I	Tinengotinib	Advanced solid tumors and TNBC	Well tolerated with preliminary activity	Active, not recruiting
NCT03195699 Phase I	Tinengotinib	Advanced solid tumors	No result available	Active, not recruiting
NCT05384119 Phase I/II	Tinengotinib + Palbociclib + Aromatase inhibitor + Fulvestrant + Ribociclib	Metastatic or locally advanced breast cancer	No result available	Completed
NCT03421353 Phase I/II	AZD9150 + Durvalumab + Cisplatin + 5-Flourouracil + Carboplatin + Gemcitabine + Nab-paclitaxel	Advanced solid tumors including TNBC	No result available	Completed

Abbreviations: triple-negative breast cancer (TNBC); Janus kinase/signal transducer and activator of transcription (JAK/STAT).

**Table 6:** Summary of clinical trials targeting PI3K pathway in TNBC.

NCT Number/Phase	Agent	Group of Patients	Result	Status
PI3K/AKT Pathway				
NCT01931163 Phase II	Everolimus + Cisplatin	TNBC post standard neoadjuvant chemotherapy	Clinical activity and good tolerability	Completed
NCT02456857 Phase II	Everolimus + Doxorubicin + Bevacizumab	Locally advanced TNBC predicted insensitive to standard chemotherapy	Safety profiles consistent with the individual agents	Completed
NCT00827567 Phase II	Everolimus	Metastatic TNBC	Terminated due to slow accrual	Terminated
NCT02531932 Phase II	Everolimus + Carboplatin	Advanced TNBC	No result available	Active, not recruiting
NCT01127763 Phase II	Everolimus + Carboplatin	Metastatic TNBC	Demonstrated clinical benefit and manageable safety after dose adjustment	Completed
NCT00930930 Phase II	Everolimus + Cisplatin + Paclitaxel + Placebo	Locally advanced TNBC	Addition of everolimus to paclitaxel/cisplatin treatment increased adverse events with no clinical benefit	Completed
NCT03805399 Phase I/II	Everolimus + Nab-paclitaxel for patients with MES TNBC subtype with PI3K/AKT pathway activation	Refractory TNBC	Promising clinical activity	Unknown status
NCT04395989 Phase II	Everolimus + Nab-paclitaxel for patients with MES TNBC subtype with PI3K/AKT pathway activation	Locally advanced or metastatic TNBC	Promising clinical activity	Active, not recruiting
NCT04251533 Phase III	Alpelisib + Nab-paclitaxel	Advanced TNBC with *PIK3CA* mutation or *PTEN* loss	Preliminary activity	Active, not recruiting
NCT04216472 Phase II	Neoadjuvant Alpelisib + Nab-paclitaxel	Anthracycline refractory TNBC	No result available	Active, not recruiting
NCT02162719 Phase II	Ipatasertib + Paclitaxel	Metastatic TNBC	Longer progression-free and overall survival	Completed
NCT02301988 Phase II	Neoadjuvant Ipatasertib + Paclitaxel	Early-stage TNBC	Longer progression-free and overall survival	Completed
NCT02423603 Phase II	Capivasertib + Paclitaxel	Advanced or metastatic TNBC	Longer progression-free and overall survival	Active, not recruiting
NCT03337724 Phase III	Ipatasertib + Paclitaxel	PIK3CA/AKT1/PTEN-altered, locally advanced or metastatic TNBC	Adding ipatasertib to paclitaxel did not improve efficacy	Completed
NCT04177108 Phase III	Ipatasertib + Atezolizumab + Paclitaxel	Locally advanced or metastatic TNBC	Adding ipatasertib did not improve efficacy	Completed
NCT04464174 Phase II	Ipatasertib + Capecitabine + Eribulin + Carboplatin + Gemcitabine	Locally advanced or metastatic TNBC	Ipatasertib + eribulin was well tolerated with encouraging effectiveness independent of PI3K/AKT status	Completed
NCT03997123 Phase III	Capivasertib + paclitaxel	Locally advanced or metastatic TNBC	Adding capivasertib to paclitaxel did not improve overall survival	Active, not recruiting

Abbreviations: triple-negative breast cancer (TNBC); phosphoinositide 3-kinase (PI3K); mesenchymal (MES).

**Table 7:** Summary of clinical trials targeting the EGFR pathway in TNBC.

NCT Number/Phase	Agent	Group of Patients	Result	Status
EGFR				
NCT00600249 Phase II	Cetuximab+ Docetaxel	Operable TNBC	Modest clinical activity with acceptable toxicity	Completed
NCT00633464 Phase II	Cetuximab + Ixabepilone	Locally advanced and/or metastatic TNBC	Addition of Cetuximab to Ixabepilone did not improve clinical activity	Completed
NCT00463788 Phase II	Cetuximab + Cisplatin	Metastatic TNBC	Addition of cetuximab to cisplatin doubled the ORR and prolonged progression-free and overall survival	Completed
NCT00894504 Phase II	Panitumumab + Gemcitabine + Carboplatin	Metastatic TNBC	Limited efficacy	Completed
NCT02593175 Phase II	Neoadjuvant Panitumumab + Carboplatin + Paclitaxel	TNBC patients which failed the doxorubicin and cyclophosphamide treatment	Pathological complete response/residual cancer burden class I = 30.2%	Active, not recruiting
NCT02876107 Phase II	Neoadjuvant Panitumumab + Carboplatin + Paclitaxel	Newly diagnosed inflammatory TNBC	No result available	Active, not recruiting
NCT01272141 Phase II	Lapatinib + Everolimus	Advanced TNBC	Slow accrual	Terminated
NCT02158507	Lapatinib + Veliparib	Metastatic TNBC	Acceptable safety profile and promising antitumor activity	Active, not recruiting
NCT03812393 Phase II	Neoadjuvant Neratinib, + Paclitaxel + Carboplatin	TNBC with enhanced HER2 signaling	Terminated due to low number of patients with no conclusive results	Terminated
NCT06008275 Phase I	Neratinib + Ruxolitinib	Chemotherapy-pretreated metastatic TNBC	No result available	Recruiting
NCT01042379 Phase II	Neratinib is one of the forty novel drugs tested	Breast cancer including a TNBC group	No result available	Recruiting

Abbreviations: triple-negative breast cancer (TNBC); human epidermal growth factor 2 (HER2); objective response rate (ORR); epidermal growth factor receptor (EGFR).

**Table 8:** Summary of clinical trials targeting the VEGFR pathway in TNBC.

NCT Number/Phase	Agent	Group of Patients	Result	Status
VEGFR				
NCT04408118 Phase II	Bevacizumab + Atezolizumab + Paclitaxel	Advanced or metastatic TNBC	Demonstrated clinical activity with acceptable safety	Completed
NCT01201265 Phase II	Bevacizumab + Gemcitabine + Carboplatin	Metastatic TNBC	Improved survival outcomes and acceptable safety profile	Completed
NCT00281697 Phase III	Bevacizumab + Paclitaxel + Gemcitabine + Vinorelbine + Capecitabine	Previously treated metastatic HER2-negative breast cancer	Improvement of progression-free survival in the TNBC group	Completed
NCT01426880 Phase II/III	Bevacizumab + Paclitaxel + Doxorubicin + Carboplatin	Early-stage breast cancer including TNBC	Enhanced pCR	Completed
NCT00567554 Phase III	Bevacizumab + Everolimus + Lapatinib + Anthracycline- and Taxane-containing chemotherapy	Primary breast cancer including TNBC	Enhanced pCR	Completed
NCT00528567 Phase III	Bevacizumab + Standard adjuvant chemotherapy	Operable primary TNBC	Did not significantly improve overall survival	Completed
NCT03797326 Phase II	Lenvatinib + Pembrolizumab	Pretreated advanced solid tumors including TNBC	Modest clinical activity and manageable safety	Completed
NCT04427293 Phase I	Lenvatinib + Pembrolizumab	Early-stage TNBC	No result available	Recruiting
NCT06140576 Phase I/II	Lenvatinib + Sindilimab + Nab-paclitaxel	Recurrent and metastatic TNBC	No result available	Not yet recruiting
NCT05007106 Phase II	Lenvatinib + Pembrolizumab + Bevacizumab + Several chemoterapeutics	Solid tumors including TNBC	No result available	Active, not recruiting
NCT00246571 Phase II	Sunitinib or standard-of-care chemotherapy	Advanced, pretreated TNBC	Inferior progression-free and overall survival compared to chemotherapy	Completed
NCT00435409 Phase III	Sunitinib + Capecitabine	Pretreated metastatic breast cancer	Addition of sunitinib to capecitabine failed to improve the clinical outcome	Completed
NCT03316586 Phase II	Cabozantinib + Nivolumab	Metastatic TNBC	Limited clinical activity	Completed
NCT03170960 Phase I/II	Cabozantinib + Atezolizumab	Advanced or metastatic solid cancers including TNBC	Clinical activity in several types of tumors but no results for TNBC	Active, not recruiting
NCT03243838 Phase II	Apatinib + Docetaxel + Epirubicin + Cyclophosphamide	Early-stage TNBC	Acceptable safety profile and high efficacy	Completed
NCT05447702 Phase II	Apatinib + Amrelizumab + Nab-paclitaxel + Epirubicin + Cyclophosphamide	Early-stage TNBC	No result available	Recruiting
NCT05556200 Phase II	Apatinib + Camrelizumab	Early-stage TNBC	No result available	Recruiting
NCT05582499 Phase II	Multiple novel drugs including Apatinib + standard chemotherapy	Operable early-stage breast cancer including TNBC	No result available	Recruiting
NCT04303741 Phase II	Apatinib + Camrelizumab + Eribulin	Advanced TNBC	Encouraging efficacy and manageable safety	Active, not recruiting
NCT03394287 Phase II	Apatinib + Camrelizumab	Advanced TNBC	Superior clinical response compared to either agent alone	Completed
NCT03945604 Phase I	Apatinib + Camrelizumab + Fuzuloparib	Recurrent or metastatic TNBC	Acceptable toxicity and preliminary antitumor activity	Completed
NCT03735082 Phase II	Apatinib + Paclitaxel + Carboplatin	Locally advanced TNBC	Addition of apatinib to chemotherapy enhanced treatment efficacy	Unknown status

Abbreviations: triple-negative breast cancer (TNBC); human epidermal growth factor 2 (HER2); pathologic complete response (pCR).

**Table 9:** Immunotherapy trials involving PD-L1 inhibition.

NCT Number/Phase	Agent	Group of Patients	Result	Status
NCT01375842 Phase I	Atezolizumab	Metastatic TNBC	Well tolerated and durable clinical benefit	Completed
NCT02425891 IMpassion130 Phase III	Atezolizumab + Nab-Paclitaxel	Previously untreated metastatic TNBC	Improved OS and PFS in treated PD-L1-positive patients	Completed
NCT03125902 Impassion131 Phase III	Atezolizumab + Paclitaxel	Previously untreated, inoperable locally advanced or metastatic, TNBC	Did not improve PFS or OS versus paclitaxel alone	Completed
NCT0319735 Impassion031 Phase III	Atezolizumab + Neoadjuvant Anthracycline/Nab-Paclitaxel-Based Chemotherapy	Primary invasive TNBC	Improved pCR rates in early-stage TNBC and acceptable safety profile	Completed
NCT04489940 Phase II	Bintrafusp alfa (M7824) antibody	High-Mobility Group AT-Hook 2 (HMGA2) expressing TNBC	Very low probability of success to justify the continuation of recruitment	Prematurely discontinued
NCT03917381 Phase I	GEN1046 (Bispecific Antibody Targeting PD-L1 and 4-1BB)	Advanced or refractory solid tumors	Manageable safety, and early clinical activity	Active, not recruiting
**Combinations with PARP Inhibition**				
NCT02734004 MEDIOLA Phase I/II	Durvalumab + Olaparib	Patients with germline *BRCA*-mutated metastatic breast cancer	Promising antitumor activity and safety similar to each agent alone	Active, not recruiting
NCT03167619 DORA Phase II	Durvalumab + Olaparib	Pretreated advanced TNBC	Longer PFS, patients with wild-type *BRCA* platinum-sensitive TNBC had durable disease control	Completed
NCT02849496 Phase II	Atezolizumab + Olaraparib	Homologous DNA repair (HDR)-deficient, locally advanced or metastatic HER2- breast cancer	No result available	Active, not recruiting
**Combinations with AKT Inhibition**				
NCT03800836 Phase Ib	Ipatasertib + Atezolizumab + Paclitaxel/Nab-Paclitaxel	Locally advanced or metastatic TNBC	Safety profile consistent with each agent’s known adverse effects	Completed
NCT04177108 IPATunity170 Phase III	Ipatasertib, Atezolizumab, and Taxane Triplet	First-line treatment for metastatic TNBC	Molecular characteristics may identify patients with favorable or unfavorable outcomes	Completed
NCT04434040 II	Ipatasertib + Atezolizumab	Residual disease in TNBC	No result available	Active, not recruiting
NCT03742102 BEGONIA Phase Ib/II	Capivasertib + Durvalumab + Paclitaxel	First-line treatment for metastatic TNBC	No result available	Active, not recruiting
**Combinations with MEK Inhibitors**				
NCT02322814 COLET Phase II	Atezolizumab + Cobimetinib + Paclitaxel	First-line treatment for metastatic or locally advanced TNBC	Safety profile consistent with known safety profiles of individual agents	Terminated due to sponsor’s decision
NCT03971409 InCITe Phase II	Avelumab + Doxorubicin + Bnimetinib	Metastatic unresectable and recurrent TNBC	No result available	Recruiting

Abbreviations: triple-negative breast cancer (TNBC); programmed cell death ligand 1 (PD-L1); human epidermal growth factor 2 (HER2); pathologic complete response (pCR); overall survival (OS); progression-free survival (PFS).

**Table 10:** Immunotherapy trials involving PD-1 inhibition.

NCT Number	Agent	Group of Patients	Result	Status
NCT01848834 KEYNOTE-012 Phase Ib	Pembrolizumab	Advanced solid tumors	Preliminary evidence of clinical activity and a potentially acceptable safety profile	Completed
NCT02447003 KEYNOTE-086 Phase II	Pembrolizumab	Previously treated metastatic TNBC	Durable antitumor activity and manageable safety profile	Completed
NCT02555657 KEYNOTE-119 Phase III	Pembrolizumab	Metastatic TNBC	No significant improvement of OS versus chemotherapy	Completed
NCT02819518 KEYNOTE-355 Phase III	Pembrolizumab + Chemotherapy	Untreated locally recurrent inoperable or metastatic TNBC	Improved OS and PFS	Completed
NCT03036488 KEYNOTE-522 Phase III	Pembrolizumab + Chemotherapy (Neoadjuvanty) and Pembrplizumab (Adjuvantly)	Early TNBC	Significant improvement of OS compared with neoadjuvant chemotherapy alone	Active, not recruiting
**Combinations with PARP Inhibition**				
NCT02657889 TOPACIO Phase I/II	Pembrolizumab + Niraparib	Advanced TNBC	Overall response rate (ORR) of 25% in platinum-resistant and ORR of 45% in patients with somatic *BRCA* mutations	Completed
NCT04191135 Phase II/III	Pembrolizumab + Olaparib	Locally recurrent inoperable or metastatic TNBC	No results available	Active, not recruiting
**Combination with VEGF Receptor Inhibition**				
NCT03394287 Phase II	Camrelizumab + Apatinib	Advanced TNBC	Higher objective response rate and manageable safety profile	Completed
**Combinations with Radiotherapy**				
NCT04837209 Phase II	Dostarlimab + Niraparib + Radiation Therapy	Metastatic PD-L1-negative or immunotherapy-refractory TNBC	No results available	Recruiting

Abbreviations: triple-negative breast cancer (TNBC); programmed cell death ligand 1 (PD-L1); overall survival (OS); progression-free survival (PFS); overall response rate (ORR).

**Table 11:** Immunotherapy trials involving CTLA-4 inhibition and next-generation immune checkpoint inhibitors.

NCT Number	Agent	Group of Patients	Result	Status
NCT02527434 Phase II	Tremelimumab	Advanced solid tumors	Suboptimal antitumor activity in BC and severe immunotherapy-related adverse effects (irAEs)	Completed
NCT01502592 Phase I	Ipilimumab	Pre-Operative, and/or cryoablation in early-stage/resectable breast cancer	Suboptimal antitumor activity in BC and severe (irAEs)	Completed
**Combinations with Other Checkpoint Inhibitors**				
NCT02536794 Phase II	Tramenitib + Durvalumab	Metastatic HER2-negative breast cancer	No results available	Terminated due to high risks in second phase
NCT02834013 Phase II	Ipilimumab + Nivolumab	Metastatic metaplastic breast cancer (MpBC)	18% overall response rate in chemotherapy-refractory MpBC	Active, not recruiting
NCT01928394 Phase I/II	Ipilimumab + Nivolumab	Advanced or metastatic solid tumors	No results reported for TNBC	Active, not recruiting
NCT03872791 Phase II	KN046 (Dual antibody targeting PD-L1 and CTLA-4) KN046 + Nab-paclitaxel	Locally advanced or metastatic TNBC	KN046 + nab-paclitaxel showed increased OS and PFS, and tolerable toxicity in the first-line treatment of metastatic TNBC, especially PD-L1-positive	Completed
**LAG-3**				
NCT03499899 Phase II	LAG525 (anti-LAG-3) + spartalizumab (anti-PD-1) LAG525 + Spartalizumab + Carboplatin LAG525 + Carboplatin	Advanced TNBC in first- or second-line therapy	No arms met the preliminary efficacy criteria. No further investigation is planned	Completed
NCT02460224 Phase I/II	LAG525 + Spartalizumab	Advanced malignancies	Well tolerated with toxicity profile of LAG525 in combination with spartalizumab comparable to spartalizumab alone. Modest antitumor activity	Completed
**TIM-3**				
NCT02608268 Phase I/II	MBG453 (anti-TIM-3) + Spartalizumab	Advanced malignancies	No results reported for TNBC	Terminated due to “Business reasons”
**TIGIT**				
NCT04584112 Phase Ib	Tiragolumab (anti-TIGIT) + Atezolizumab + Chemotherapy	Early and metastatic TNBC	No results available	Completed
**VISTA**				
NCT02671955 Phase II	JNJ-61610588 (anti-VISTA)	Advanced malignancies	No results reported for TNBC	Terminated due to “Business decision”
NCT04475523 Phase I	CI-8993 (anti-VISTA)	Advanced Solid Tumors	No results reported for TNBC	Completed
NCT02812875 Phase I	CA-170 (anti- PD-L1/PD-L2/VISTA)	Advanced solid tumors and lymphomas	No results reported for TNBC	Completed

Abbreviations: triple-negative breast cancer (TNBC); programmed cell death ligand 1 (PD-L1); programmed cell death protein 1 (PD-1); human epidermal growth factor 2 (HER2); pathologic complete response (pCR); overall survival (OS); progression-free survival (PFS); metastatic metaplastic breast cancer (MpBC); breast cancer (BC).

**Table 12:** Immunotherapy trials involving modulation of tumor microenvironment.

NCT Number	Agent	Group of Patients	Result	Status
Targeting Tumor Associated Macrophages				
NCT04331067 Phase Ib/II	Cabiralizumab (anti-CSF1R) + Nivolumab (anti-PD-1) + neoadjuvant chemotherapy	Localized TNBC	No results reported	Active, not recruiting
**Adenosine Pathway Inhibition**				
NCT03207867 Phase II	NIR178 (anti-A2aR) + Spartalizumab (anti-PD-1 PDR001)	Solid tumors and Non-Hodgkin lymphoma	Clinical benefit in a fraction of patients with relapsed TNBC	Terminated due to “Sponsor decision”
NCT03742102 BEGONIA Phase Ib/II	Oleclumab (anti-CD73)+ Durvalumab (anti- PD-L1) + Paclitaxel	First-line Metastatic TNBC	Safety profiles consistent with the individual agents	Active, not recruiting
NCT03629756 Phase I	AB928, (selective A_2a_R/A_2b_R antagonist) + zimberelimab (AB122) (anti-PD-1)	Advanced malignancies	Favorable safety profile of AB928 combination therapy	Completed
**Cytokine Based Therapies**				
KEYNOTE-890 trial (NCT03567720) Phase II	intratumoral electroporation of tavokinogene telseplasmid encoding p35 and p40 subunits of human interleukin 12 + Pembrolizumab	Inoperable locally advanced or metastatic TNBC	Induced responses in treated and contralateral tumors	Active, not recruiting
NCT02869295 Phase I	NKTR-214 (PEGylated interleukin-2)	Locally advanced or metastatic solid tumors	Biological activity and good tolerability	Completed
NCT02983045 Phase I/II	NKTR 214 + Nivolumab	Selected advanced solid tumors	No results reported for TNBC	Completed
NCT03424005 Phase I/II	NKTR-214 + Atezolizumab (anti-PDL-1)	Metastatic or inoperable locally advanced TNBC patients: PD-L1-positive without prior therapy disease progression after treatment with chemotherapy	No results available	Recruiting
**Toll-Like Receptor Agonists**				
NCT04916002 Phase II	CMP-001 (virus-like particle containing TLR9 agonist) + Cemiplimab (anti-PD-1)	Selected types of advanced or metastatic solid tumors	No reported results	Terminated Due to “Sponsor Decision related to study drug supply“
NCT01042379 I-SPY Phase I	SD-101 (TLR-9 agonist) + Pembrolizumab	Breast cancer	No results reported for TNBC	Recruiting
NCT03435640 368 REVEAL Phase II	NKTR-262 (TLR 7/8 agonist) + NKTR-214 + Nivolumab	Locally advanced or metastatic solid tumors	Enhanced antigen presentation and costimulatory activity	Terminated by the sponsor based on the overall results from the phase I part of the study

Abbreviations: triple-negative breast cancer (TNBC); programmed cell death ligand 1 (PD-L1); programmed cell death protein 1 (PD-1).
